# Overcoming BET‐inhibitor JQ1 resistance in aggressive non‐small cell lung cancer by inducing ferroptosis via inhibition of the BRD2–FTH1 axis

**DOI:** 10.1111/febs.70191

**Published:** 2025-07-13

**Authors:** Stefania Scicchitano, Cinzia Garofalo, Beatrice Stella, Gianluca Santamaria, Flora Cozzolino, Vittoria Monaco, Flavia Biamonte, Maria Monti, Eleonora Vecchio, Maria Concetta Faniello

**Affiliations:** ^1^ Department of Experimental and Clinical Medicine Magna Graecia University of Catanzaro Italy; ^2^ Department of Chemical Sciences Federico II University of Naples and CEINGE Advanced Biotechnologies Franco Salvatore Italy

**Keywords:** BRD2, ferroptosis, FTH1, JQ1, lung cancer, oxidative damage

## Abstract

Recent studies emphasize the involvement of the nuclear H ferritin subunit (FTH1; also known as ferritin heavy chain) in DNA protection from oxidative damage and transcriptional regulation. Bromodomain and extra‐terminal domain (BET) proteins act as epigenome readers for transcriptional regulation. Among them, the role of bromodomain‐containing protein 2 (BRD2) in non‐small cell lung carcinoma (NSCLC) remains unclear. Moreover, the clinical utilization of BET bromodomain inhibition is severely limited by different sensitivities among NSCLC subtypes. This study provides the first evidence of nuclear BRD2‐FTH1 functional interplay. Nuclear FTH1 is associated with BRD2, not bromodomain‐containing protein 4 (BRD4), in a panel of NSCLC cell lines and affects BRD2 protein stability only in more aggressive types of NSCLC cells. In addition, the protective function of FTH1 was abrogated in *FTH1‐*silenced cells that are resistant to synthetic BET bromodomain inhibitor JQ1 (a thieno‐triazolo‐1,4‐diazepine) but not in JQ1‐sensitive cells, leading to an increase in mortality. Then, the potential mechanism by which the combination of JQ1 with *FTH1* silencing induces cell death was explored. The results show that ferroptosis is involved in the anticancer effect of JQ1 upon *FTH1* silencing only in JQ1‐insensitive cells. Moreover, the expression of ferroptosis‐associated genes glutathione peroxidase 4 (*GPX4*), solute carrier family 7 member 11 (*SLC7A11*) and solute carrier family 3 member 2 (*SLC3A2*) was downregulated under JQ1 treatment only after *FTH1* silencing, indicating that the BRD2 inhibition due to the co‐treatment could regulate the expression of ferroptosis‐associated genes. In summary, for the first time, our data suggest that *FTH1* silencing may serve as an effective anti‐tumor strategy to enhance the activity of JQ1, acting to overcome the chemotherapy resistance in more aggressive NSCLCs.

## Graphical abstract

Recent studies emphasize alternative functions of FTH1 relevant for nuclear processes, such as transcriptional regulation. This study shows a novel FTH1/BRD2 association in non‐small cell lung carcinoma (NSCLC). This nuclear interaction affects BRD2 protein stability in aggressive NSCLC cells. FTH1 depletion sensitizes resistant cells to BET inhibitor JQ1, inducing ferroptosis. The combination treatment of JQ1 and FTH1 silencing suggests a potential anti‐tumor strategy to overcome chemotherapy resistance in aggressive NSCLC.

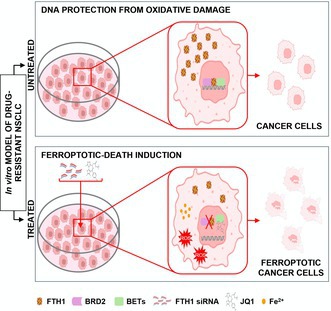

**Abbreviations**
Ac: acidic region
ALADIN: Alacrima–Acalasia–Adrenal insufficiency neurological disorder
AML: acute myeloid leukemia
BET: bromodomain and extra‐terminal domain
BRD2: bromodomain‐containing protein 2
BRD4: bromodomain‐containing protein 4
CM‐H2DCFDA: 2′‐7′‐dichlorodihydrofluorescein diacetate
CPTAC: Clinical Proteomic Tumor Analysis Consortium
CRYAB: alpha B crystallin
DAXX: death domain‐associated nuclear protein
DFO: deferoxamine
DFX: deferasirox
ET: extra‐terminal domain
FBS: fetal bovine serum
Fe^2+^
: reduced ferrous
Fe^3+^
: oxidized ferric
Fer‐1: ferrostatin
FTH1: ferritin heavy chain subunit
FTL: ferritin light chain
GPX4: glutathione peroxidase 4
IP: immunoprecipitation
JQ1: a thieno‐triazolo‐1,4‐diazepine
LIP: labile iron pool
LUAD: lung adenocarcinoma
mD: motif B
METTL14: methyltransferase‐like 14
MFI: mean fluorescence intensity
MitoSOX Red: mitochondrial superoxide indicator
MOC: Manders' overlap coefficient
NMC: NUT midline carcinoma
NSCLC: non‐small cell lung carcinoma
SLC3A2: solute carrier family 3 member 2 (SLC3A2)
SLC7A11: solute carrier family 7 member 11
YAP: yes‐associated protein

## Introduction

Iron is an essential element for cell life, involving many metabolic routes including cell replication and cell growth. On the other hand, it is potentially toxic since it can easily shuttle between the reduced ferrous (Fe^2+^) and the oxidized ferric (Fe^3+^) forms, thus contributing to profound alterations in the redox balance of the cell. Ferritin, by storing iron atoms in the Fe^3+^ non‐toxic form, is the key protein in maintaining intracellular iron homeostasis [1, 2]. The molecule is composed of 24 subunits of heavy (FTH1) and light (FTL) type assembled to form a shell with a central cavity where the iron is stored; the FTH1 subunit, in addition to participating in the constitution of the shell, is provided with a ferroxidase activity, essential to allow the iron to be conserved in the ferric status. Ferritin molecules are distributed in the cytoplasm, mitochondria, and nuclei [3]; the cytoplasmic form is a heteropolymer of heavy and light types, while nuclear ferritin is prevalently composed of FTH1 subunits [4, 5, 6]. Nuclear ferritin is preferentially associated with heterochromatin and has been described in corneal epithelial cells, macrophages, hepatocytes, reticular, muscle, and nerve cells, and also in some brain tumors and glial cell lines *in vitro* [7]. So far, the role of nuclear ferritin is still poorly defined. It might be involved in enabling DNA synthesis [8]. Nuclear ferritin binds to DNA and protects it from UV and iron‐induced oxidative stresses, through the sequestration of excess iron [4, 5, 6]. Noteworthy, a DNA‐binding motif in nuclear ferritin raises the possibility to act as gene regulator to repress the human adult β‐globulin gene in embryonic erythroid cells [9]. Indeed, in cancer cells, reduced levels of nuclear ferritin increase their sensitivity to radiation and chemotherapy [10, 11]. These reports confer a moonlight function to FTH1 as an iron storage protein and in the maintenance of redox balance but also relevant for nuclear processes, as transcriptional regulation.

The bromodomain and extra‐terminal domain (BET) family of proteins consists of BRD2, BRD3, and BRD4 somatic members, and of the testis‐specific BRDT. These proteins act as epigenetic transcription regulators by binding histones and finally by recruiting co‐activators or co‐repressors to specific gene promoters. As epigenetic readers modulating the transcription of several cancer‐related genes, BET proteins are considered potential therapeutic targets in many cancer types, such as acute myeloid leukemia (AML) [12] and NUT midline carcinoma (NMC) [13, 14], and the identification of inhibitory mechanisms and/or drugs able to suppress BET pathways is currently a hot topic in cancer research. BET family inhibitors competitively bind to the acetyl‐lysine recognition pockets of BET proteins, resulting in the release from active chromatin and the suppression of downstream signaling to RNA polymerases [15]. JQ1 (thieno‐triazolo‐1,4‐diazepine) is the first described pan‐BET inhibitor that mimics acetylated lysine, binding competitively and specifically to the BD1 and BD2 bromodomains with high affinity [13]. JQ1 shows antiproliferative effects against NUT midline carcinoma (NMC), medulloblastoma, and breast and lung cancer [16, 17, 18]. In addition to JQ1, the pan‐BET inhibitor OTX015 exerts anticancer activity in hematological cancers and some solid cancer types such as mesothelioma and neuroblastoma [19, 20, 21]. Recently, I‐BET762 has shown anticancer activities in neuroblastoma, pancreatic preclinical cancer models [22, 23], NSCLC, triple‐negative breast cancer, and gastrointestinal stromal cancer [24, 25]. However, cancer cells may show inherited resistance or may gradually escape apoptosis caused by BET inhibitors such as JQ1, leading to chemotherapy failure [26, 27]. A deeper understanding of the molecular mechanisms underlying BETi resistance is necessary to improve the effectiveness of this chemotherapeutic approach.

Recent research from lung cancer models on BET inhibitors efficacy has been published [28]. Different from blood tumors, a subset of lung adenocarcinoma cell lines is acutely susceptible to the JQ1 BET inhibitor, through a mechanism independent of *MYC* downregulation [28].

Non‐small cell lung cancer (NSCLC) is the leading cause of cancer‐related death worldwide [29]. NSCLC, including adenocarcinoma, squamous cell carcinoma, and large cell carcinoma, accounts for approximately 83% of lung cancers [30]. Because most patients are often diagnosed at late stages with local invasion or metastasis, combined treatment including chemotherapy and molecular‐targeted therapy is commonly used to ameliorate patient's outcome [30]. To date, the effect and mechanism of BET inhibition have not been sufficiently clarified in lung cancer. Therefore, identifying the downstream signals that mediate its effects in different contexts and elucidating the mechanism are urgently needed. In this study, we investigated the effects of JQ1 inhibition in unresponsive NSCLC cells and discovered, for the first time, a nuclear interaction BRD2/FTH1 that led to re‐sensitization using a combination approach with *FTH1* silencing. The combination of JQ1/*FTH1* silencing promoted ferroptosis, reducing tumor growth in cells that showed a poor response to JQ1 alone. The present findings could provide new strategies for enhancing the anticancer effects of the JQ1‐BET inhibitor.

## Results

### FTH1 interacts with BRD2 within nuclei in NSCLC cells

**Fig. 1:**
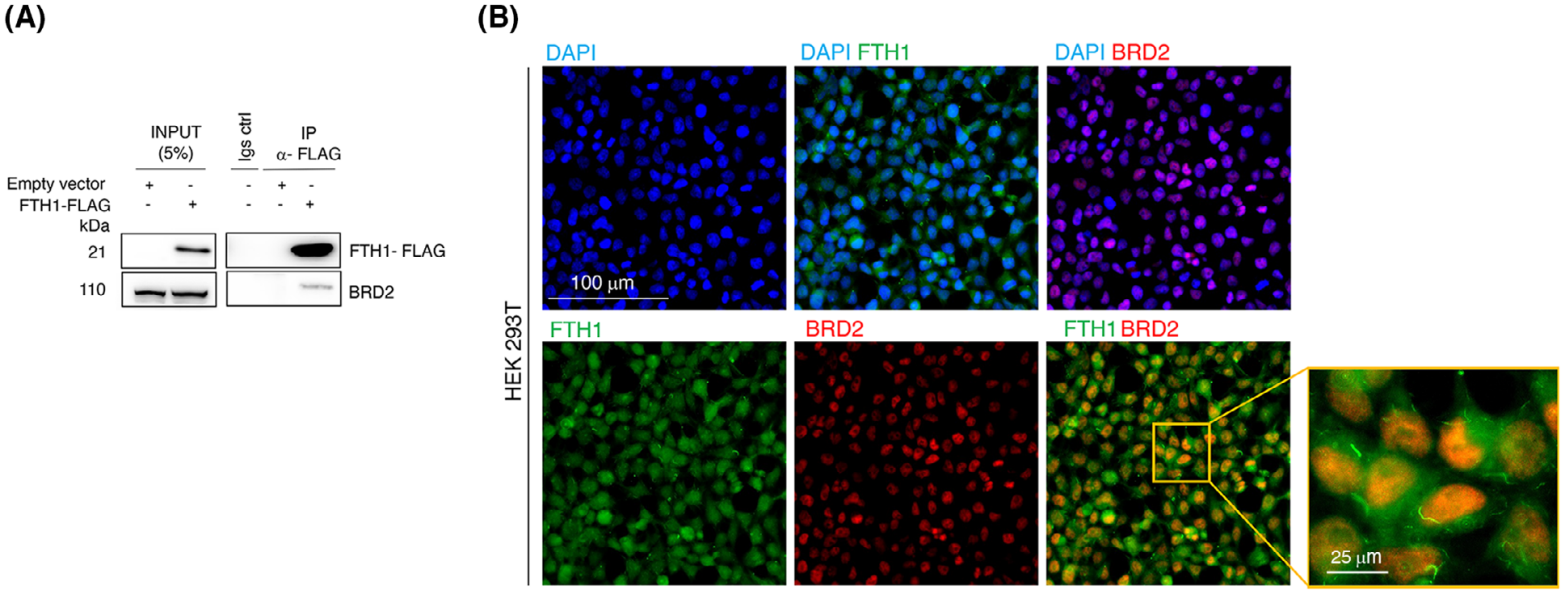
Ferritin heavy chain interacts with BRD2 in HEK 293T. (A) HEK293T cells (3 × 10^6^) were transfected with 3xFLAG‐FTH1 or 3xFLAG (empty vector) (4 μg). Cell extracts were immunoprecipitated via incubation with protein G‐Sepharose coupled to anti‐FLAG antibody; immunocomplexes were separated by SDS/PAGE and analyzed by western blotting using the anti‐FTH1 and anti‐BRD2 antibodies. We use the symbols ‘+’ or ‘−’ to indicate the presence or the absence of each vector, respectively. A representative image from three independent experiments is shown. (B) HEK‐293T cells were grown on a coverslip, fixed with 4% paraformaldehyde, and processed for double‐label immunofluorescence with anti‐FTH1 (green) and anti‐BRD2 (red) antibodies. DAPI (blue) was used for nuclei staining. Images were collected using the Thunder microscopy system (40×); scale bar 100 μm and scale bar 25 μm for magnified images.

**Fig. 2:**
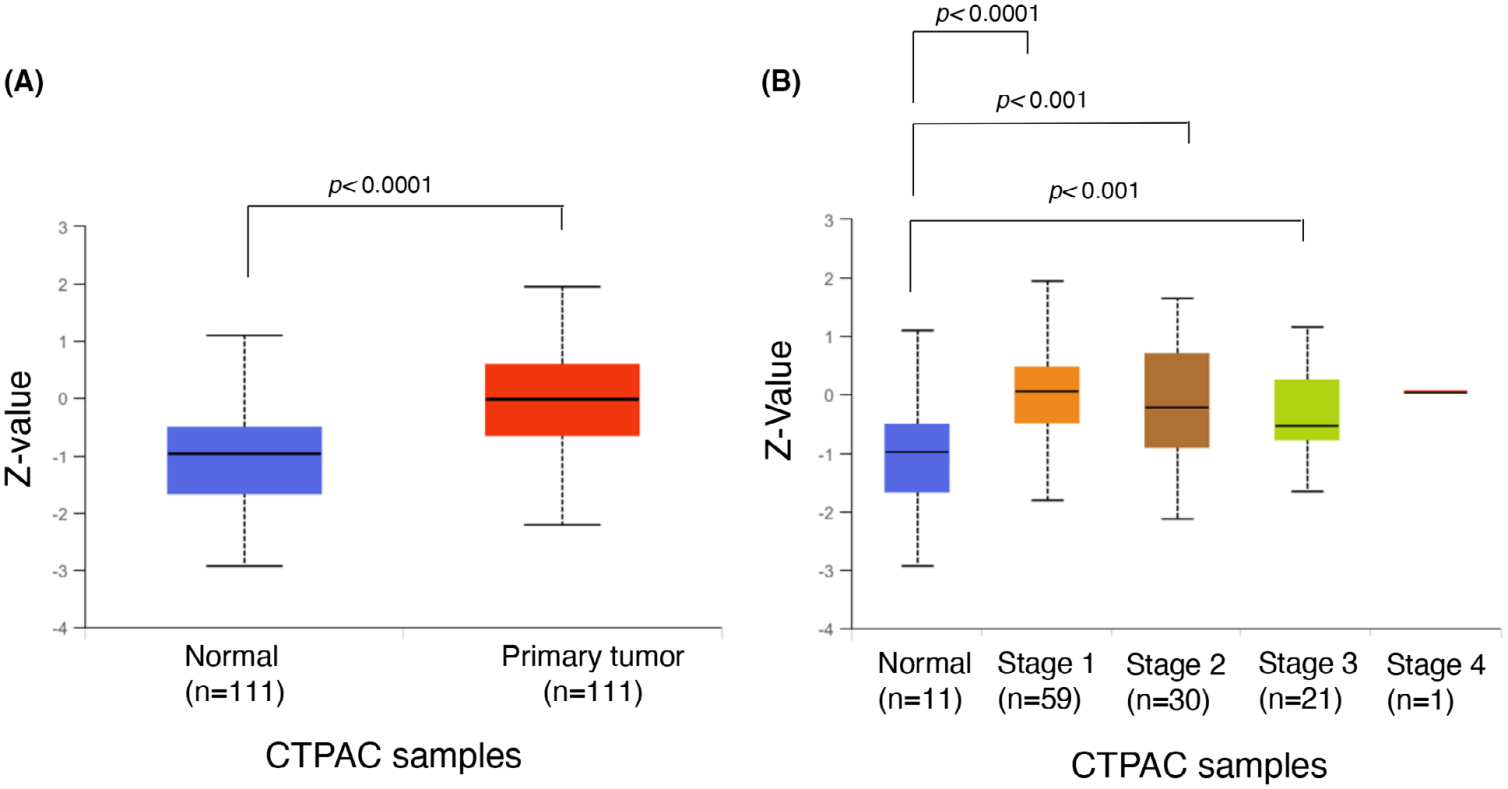
Expression of BRD2 in cancer patients included in The Cancer Genome Atlas (TGCA) database. (A) A boxplot of the BDR2 protein expression values between normal and primary tumor patients (*P* value = 1.43E‐14). *Z*‐values represent standard deviations (SD) from the median across samples. This data incorporates the mass spectrometry data generated from TCGA samples by the CPTAC consortium. Data were statistically analyzed by Student's *t* test. (B) Boxplot showing protein expression of BDR2 in normal individuals and in LUAD patients stratified by cancer stages. Z‐values represent standard deviations (SD) from the median across samples. This data incorporates the mass spectrometry data generated from TCGA samples by the CPTAC consortium Data were statistically analyzed by Student's *t* test.

**Fig. 3:**
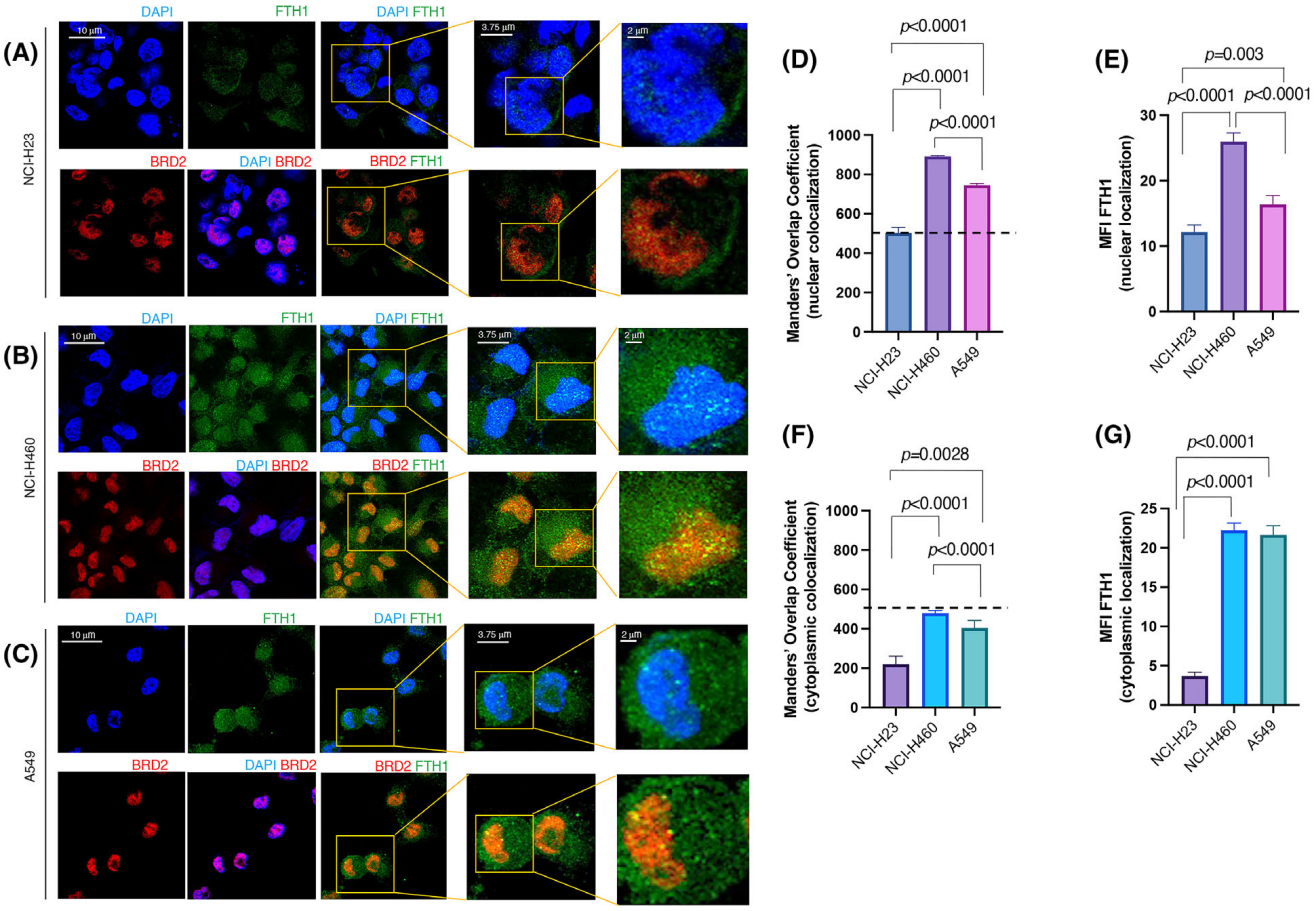
Ferritin heavy chain interacts with BRD2 in a panel of NSCLC cells. (A) NHI‐H23, (B) NHI‐H460, (C) A549 cells were grown on a coverslip, fixed with 4% paraformaldehyde, and processed for double‐label immunofluorescence with anti‐FTH1 (green) and anti‐BRD2 (red) antibodies. DAPI (blue) was used for nuclei staining. Single Z stack images were collected using a Leica Stellaris 5 Confocal Microscope (63×); scale bar 10 μm, and scale bar 3.75–2 μm for magnified images. Representative images are shown. (D, E) Graph showing quantification of FTH1 nuclear/cytoplasmic localization intensity as MFI (mean of fluorescence intensity) in NHI‐H23, NHI‐H460, and A549 cell lines. The quantification rate was performed with > 60 cells examined per cell line from three different experiments. (F, G) Graphs showing levels of FTH1 and BRD2 co‐localization in NHI‐H23, NHI‐H460, and A549 cell lines in nuclei and cytoplasmic areas, respectively, using Manders' overlap coefficient (MOC). MOC represents the percentage of BRD2 (red) that colocalized with FTH1 (green). The horizontal dashed line indicates the cut‐off of 50%. The quantification rate was performed with > 60 cells examined per cell line from three different experiments. For panels (D–G) all results are mean ± SEM from three representative experiments. Data were statistically analyzed by Student's *t* test.

Using a functional proteomics approach relied on a combination of immunoprecipitation and mass spectrometry, we have previously identified BRD2 as one of the FTH1 interactors [31]. In this work, we first verified the physical interaction between the FTH1 and BRD2 in HEK293T vector cells transfected with the expression vector 3xFlag‐FTH1 or the empty vector 3xFlag by performing co‐immunoprecipitation assays. We found that BRD2 co‐immunoprecipitated with FTH1‐FLAG using anti‐FLAG antibody (Fig. 1A). This result was confirmed by immunofluorescence analysis, which further highlighted that the BRD2/FTH1 complex localized within HEK293T nuclei (Fig. 1B). Previous studies have reported that overexpression of BRD2 occurs in human cancers [14, 32, 33, 34]. Still, studies about lung cancer are minimal. We sought to evaluate the expression pattern of BRD2 by using the Clinical Proteomic Tumor Analysis Consortium (CPTAC) for the lung adenocarcinoma dataset. We discovered that in a cohort of primary lung adenocarcinoma (LUAD) patients (*n* = 111), BRD2 expression was higher than in healthy subjects (*P* < 0.0001) (Fig. 2A). Furthermore, among different stages of LUAD, BRD2 expression was higher in stages I (*P* < 0.0001), II (*P* < 0.001), and III (*P* < 0.001) than in their normal counterparts (Fig. 2B). In summary, BRD2 expression is higher in LUAD tissues than in normal tissues and is positively correlated with different stages of cancers. Next, we moved our analyses to a panel of non‐small cell lung cancer (NSCLC) cell lines (NCI‐H23, NCI‐H460, and A549). Of note, all NSCLC cell lines tested harbor *KRAS* mutations, and two of three cell lines (NCI‐H460, and A549) have concurrent *KRAS* and *LKB1* mutations [28, 35, 36, 37, 38]. We confirmed both the BRD2‐FTH1 interaction and the nuclear co‐localization of the BRD2‐FTH1 complex through immunofluorescence analysis in all three NSCLC cell lines (Fig. 3A–C). Furthermore, we noticed that the percentage of the co‐localization of BRD2‐FTH1 in the nuclei was significantly higher in NCI‐H460 cells compared to A549 cells (*P* < 0.0001) and to NCI‐H23 (*P* < 0.0001), respectively, being, in particular, almost 2‐fold higher in NCI‐H460 cells compared to NCI‐H23 cells, where the Manders' overlap coefficient (MOC) percentage is under the cut‐off of 50% that is considered no co‐localization (Fig. 3D). Interestingly, this was in line with the different FTH1 nuclear quantities among the three cell lines, with the highest in NCI‐H460 cells compared to A549 (*P* < 0.0001) and the lowest in NCI‐H23 cells compared to NCI‐H460 cells (*P* < 0.0001) (Fig. 3E). We proceeded to quantify the degree of co‐localization of BRD2‐FTH1 in the cytoplasm. In all three cell lines, the MOC percentage per cell line was below 50% (Fig. 3F). On the other hand, the cytoplasmic localization of FTH1 seemed higher in NCI‐H460 and A549 than in NCI‐H23 (*P* < 0.0001 in both cases; Fig. 3G). As already mentioned, BRD2 is one of the members of the BET domain‐containing proteins along with the most studied BRD4. In order to assess whether FTH1 specifically may interact with other members of BET domain‐containing proteins or, rather, specifically interact only with BRD2, we performed immunofluorescence co‐localization assays between BRD4 and FTH1. As shown in Fig. S1A–C, no co‐localization was observed in the nuclei between the two proteins in any of the three NSCLC cell lines, as confirmed by Manders' overlap coefficient calculation (Fig. S1D). In addition, we noticed that differently from BRD2, BRD4 showed a prevalent cytoplasmic distribution in the three cell lines analyzed. Overall, these results indicate for the first time that FTH1 specifically interacts with BRD2 within the nuclei of NSCLC cells.

### BRD2‐FTH1 interaction modulates BRD2 protein amounts

**Fig. 4:**
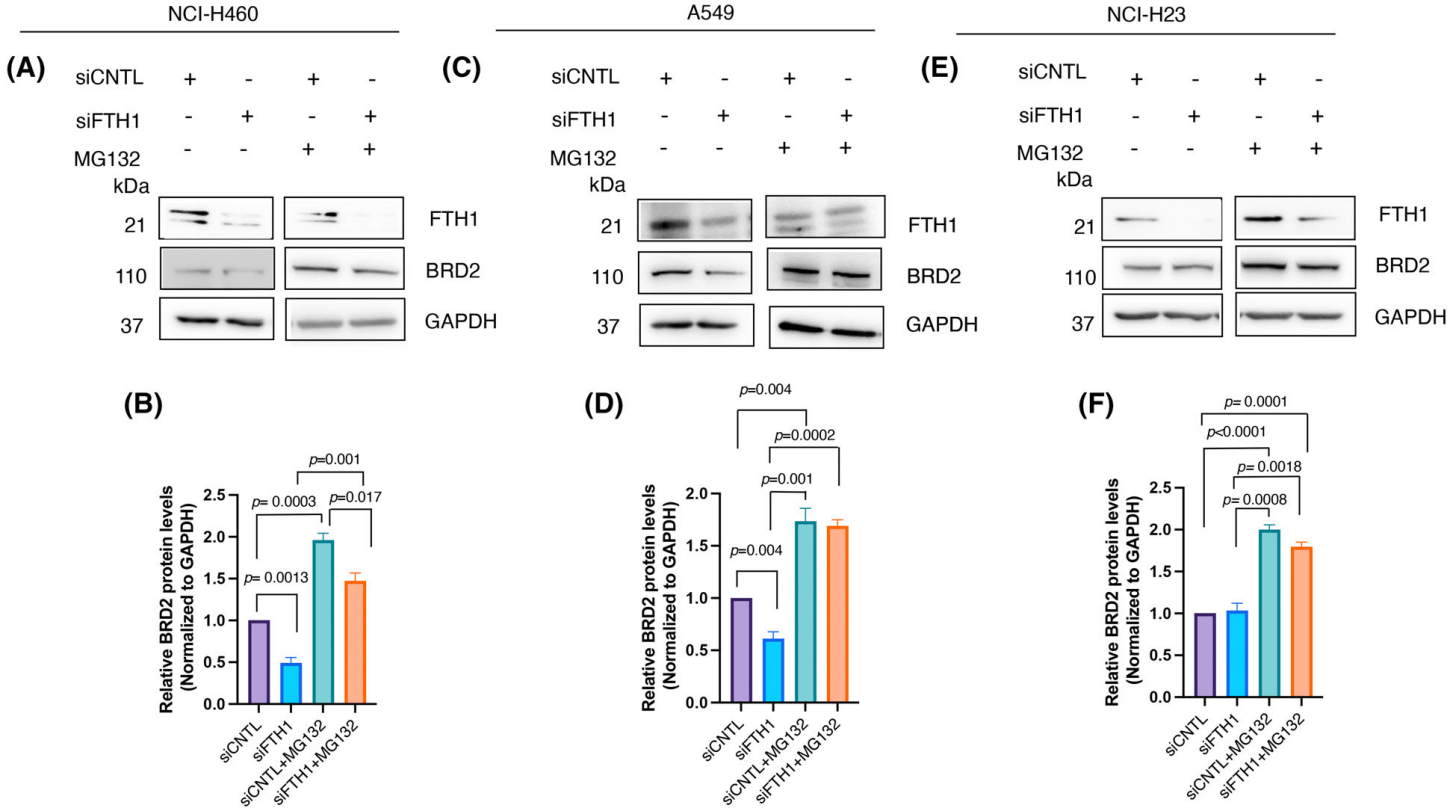
Ferritin heavy chain is involved in BRD2 protein stability in NSCLC JQ1‐resistant. NCI‐H23, NCI‐H460, and A549 (3 × 10^6^) were transiently transfected with a pool of siFTH1 or siRNA control and treated or not with the proteasome inhibitor MG132 (40 μm) for 5 h, then lysed 48 h later. Whole cell extracts (30 μg) of siFTH1 or siCNTL and siFTH1 or siCNTL treated or not with MG132 (A) NCI‐H23, (C) NCI‐H460, and (E) A549 cells, respectively, were separated by 12% SDS/PAGE and analyzed by western blotting using anti‐FTH1, anti‐BRD2, and anti‐GAPDH antibodies. We use the symbols ‘+’ or ‘−’ to indicate the presence or the absence of MG132, respectively. We use the symbols ‘+’ or ‘−’ to indicate the presence or the absence of each vector, respectively. In (B, D, F) panels, densitometric analysis was performed. Densitometric values of BRD2 bands were normalized to GAPDH bands. Values (mean ± SEM, *n* = 3) are shown. Statistically significant difference was calculated according to Student's *t* test.

As a result of their physical interaction with FTH1, some proteins appeared modulated in their quantity and activity (i.e., the tumor suppressor p53) [39] or only in their activity (i.e., the chemokine receptor CXCR4) [40]. Here, we sought to explore the effects exerted by FTH1 interaction on BRD2 stability/amount. To achieve this, we transiently transfected NCI‐H23, NCI‐H460, and A549 cells with a pool of siFTH1 and analyzed the BRD2 protein content. As shown by western blotting analysis, *FTH1* silencing was accompanied by a consistent decrease of BRD2 protein amount in NCI‐H460^siFTH1^ and A549^siFTH1^ cells, where it appears halved (Fig. 4A–D) rather than NCI‐H460^siCNTL^ and A549^siCNTL^ cells, respectively; unlike that, no changes occurred in NCI‐H23^siFTH1^ cells rather than NCI‐H23^siCNTL^ (Fig. 4E,F). The results were consistent with the different rates of nuclear co‐localization of the two proteins among the cell lines. We inquired ourselves: Could the decrease of BRD2 levels be connected to the control of its nuclear concentration? In this scenario, we hypothesize that FTH1 could be responsible for nuclear BRD2 stability. FTH1 depletion in the cells, hence in the nuclei, could trigger BRD2 transfer to the cytosol, where it can undergo proteasomal degradation. To solve this question, we transiently transfected NCI‐H23, NCI‐H460, and A549 cells with a pool of siFTH1 and treated cells with MG132, a 26S proteasome inhibitor, to analyze the BRD2 protein content. As shown by western blotting analysis, the presence of the proteasome MG132 restored the BRD2 protein amount in NCI‐H460 and A549 cells (Fig. 4A–D), respectively, while the BRD2 amount remained unaffected in NCI‐H23 cells (Fig. 4E,F). To assess any possible effect at mRNA levels, we performed gene expression analysis of *BRD2* in *FTH1*‐silenced and not‐silenced NSCLC cell lines. As shown in Fig. S2A–C, *BRD2* mRNA steady‐state amounts were unaffected by FTH1 knockdown. These results indicate that FTH1 could be involved in the proteasomal degradation of BRD2.

### Interfering with BRD2‐FTH1 interaction counteracts the resistance of NCI‐H460 cells to JQ1

**Fig. 5:**
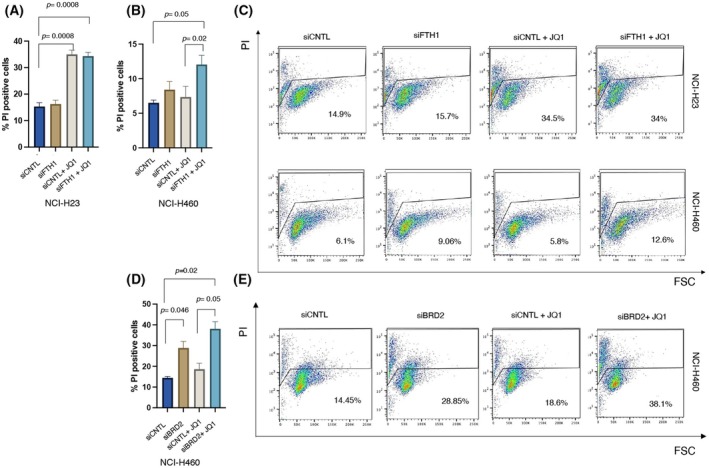
*FTH1* silencing combined with BET inhibitor JQ1 increases cell death of NCI‐H460 JQ1‐insensitive cells. The same effect is observed when *BRD2* is knocked down. Cells (3 × 10^6^) were transiently transfected with a pool of siFTH1 (NCI‐H460, NCI‐H23) or siBRD2 (NCI‐H460) or siRNA control. Twenty‐four hours later, transfected cells were treated or not with JQ1 and lysed 24 h later. (A, B, and D) panels show Bar diagram of the percentage of death cells (PI‐Propidium Iodide‐positive cells) as measured by flow cytometry. Values (mean ± SEM; *n* = 3) are shown. Statistically significant difference was determined by Student's *t* test. (C) Representative density plots of PI staining per forward scatter (FSC; size) of NCI‐H460 (up panel), and NCI‐H23 (down panel) cells transiently transfected with a pool of siFTH1or siRNA control and treated or not with JQ1 from three independent experiments is shown. (E) Representative density plots of PI staining per forward scatter (FSC; size) of NCI‐H460 cells transiently transfected with a pool of siBRD2 or siRNA control and treated or not with JQ1 from three independent experiments is shown.

BET inhibitors JQ1, OTX015, and I‐BET62 [15, 17, 41, 42, 43] are currently used as therapeutic approaches in a wide spectrum of human cancer. JQ1 may exert its antiproliferative effect by suppressing BRD2 in solid tumors, including NSCLC, where, though, its clinical utilization is severely limited by a different sensitivity among cancer cell subtypes [28, 44, 45, 46]. Of note, antiproliferative and pro‐apoptotic activity of BET inhibition was observed in NSCLC cells bearing a mutation in *KRAS* [28, 35, 41]. Otherwise, NSCLC cells harboring concurrent *LKB1* and *KRAS* mutations displayed resistance to bromodomain inhibition [35, 37]. Given that the presence of the BRD2‐FTH1 interaction affects the intracellular amount of BRD2, we wondered if disrupting the BRD2‐FTH1 complex could alter NSCLC cells' response to the prototype BET inhibitor, JQ1. NCI‐H460 (*KRAS/LKB1* mutant) cells were selected as NSCLC cells showing the highest rate of BRD2‐FTH1 co‐localization and resistance to JQ1 [37, 41, 47]. Conversely, NCI‐H23 (*KRAS* mutant) cells displayed the lowest BRD2‐FTH1 co‐localization and the most sensitivity to JQ1 [41, 47]. However, previous findings described the antiproliferative activity of BET inhibitors in sensitive cells with cell cycle arrest and induction of apoptosis in lung adenocarcinoma and myeloma cell lines [28, 41, 48]. Chiefly, we calculated the half inhibitory concentration (IC_50_) values for JQ1 on NCI‐H23 and NCI‐H460 cells (Fig. S3A). They are listed in Fig. S3B and ranged from 4 to 12.6 μm. The IC_50_ value for NCI‐H460^siCNTL^ was not determinable, since JQ1 appeared to be virtually ineffective on these cells at indicated concentrations. In line with these data and previous literature [28, 41], from now on, JQ1 has been employed at the concentration of 10 μm for 24 h. Then, we transiently silenced *FTH1* in NCI‐H23 and NCI‐H460 cell lines and assessed cell death upon treatment with JQ1. Both cell lines were tested for the reduction of FTH1 mRNA levels, as illustrated in Panels A and D of Fig. S4. The results of propidium iodide (PI) staining indicate that NCI‐H23^siFTH1^ cells did not change the sensitivity to JQ1, as shown by the comparable percentage (34.97% vs. 34.33%; *P* > 0.05, Fig. 5A,C) of PI^+^ cells upon JQ1 treatment alone (*P* = 0.0008) or in combination with *FTH1* depletion (*P* = 0.0008) (Fig. 5A,C). These results were also confirmed by both trypan blue dye exclusion and Cell Titer‐Glo Luminescent assays (Fig. S4B,C). In NCI‐H460 cells, however, while JQ1 treatment alone was completely ineffective (*P* > 0.05), the combined use of JQ1 with *FTH1* silencing triggered cytotoxicity compared to JQ1 alone (*P* = 0.02) or NCI‐H460^siCNTL^ cells (*P* = 0.05) (Fig. 5B,C). No alterations were observed in the two cell lines transiently transfected with siFTH1 alone (Fig. 5A–C). These results were also confirmed by both trypan blue dye exclusion and Cell Titer‐Glo Luminescent assays (Fig. S4E,F). Finally, we inquired about the possibility of *BRD2* knockdown counteracting NCI‐H460 resistance to JQ1. As shown in Panels D and E of Fig. 5, we found that, similarly to *FTH1* knockdown, *BRD2* silencing in combination with JQ1 treatment induced 38.1% of PI^+^ cells compared to JQ1 alone (18.6%; *P* = 0.05; Fig. 5D,E). In addition, unlike *FTH1* knockdown, the *BRD2* silencing can induce an around 2‐fold increase in the percentage of cell death of NCI‐H460 cells (28.85% vs. 14.45%; *P* = 0.046; Fig. 5C,E). These results were also confirmed by both trypan blue dye exclusion and Cell Titer‐Glo Luminescent assays (Fig. S4G,H). Also, for *BRD2* silencing, the NCI‐H460 cell line was tested for the reduction of BRD2 mRNA levels, as illustrated in Fig. S4I. Overall, these results indicate that *FTH1* silencing is able to lead to antiproliferative effects in NCI‐H460 cells upon treatment, overcoming the JQ1 resistance. Moreover, our data suggest that the increased vulnerability to JQ1 in NCI‐H460 is likely due to the BRD2‐FTH1 interaction, as in the same experimental conditions, BRD4 protein or mRNA levels remain unaffected by *FTH1* depletion (Fig. S5A–C).

### *FTH1* silencing in combination with JQ1 induces ferroptosis in NCI‐H460 cells

**Fig. 6:**
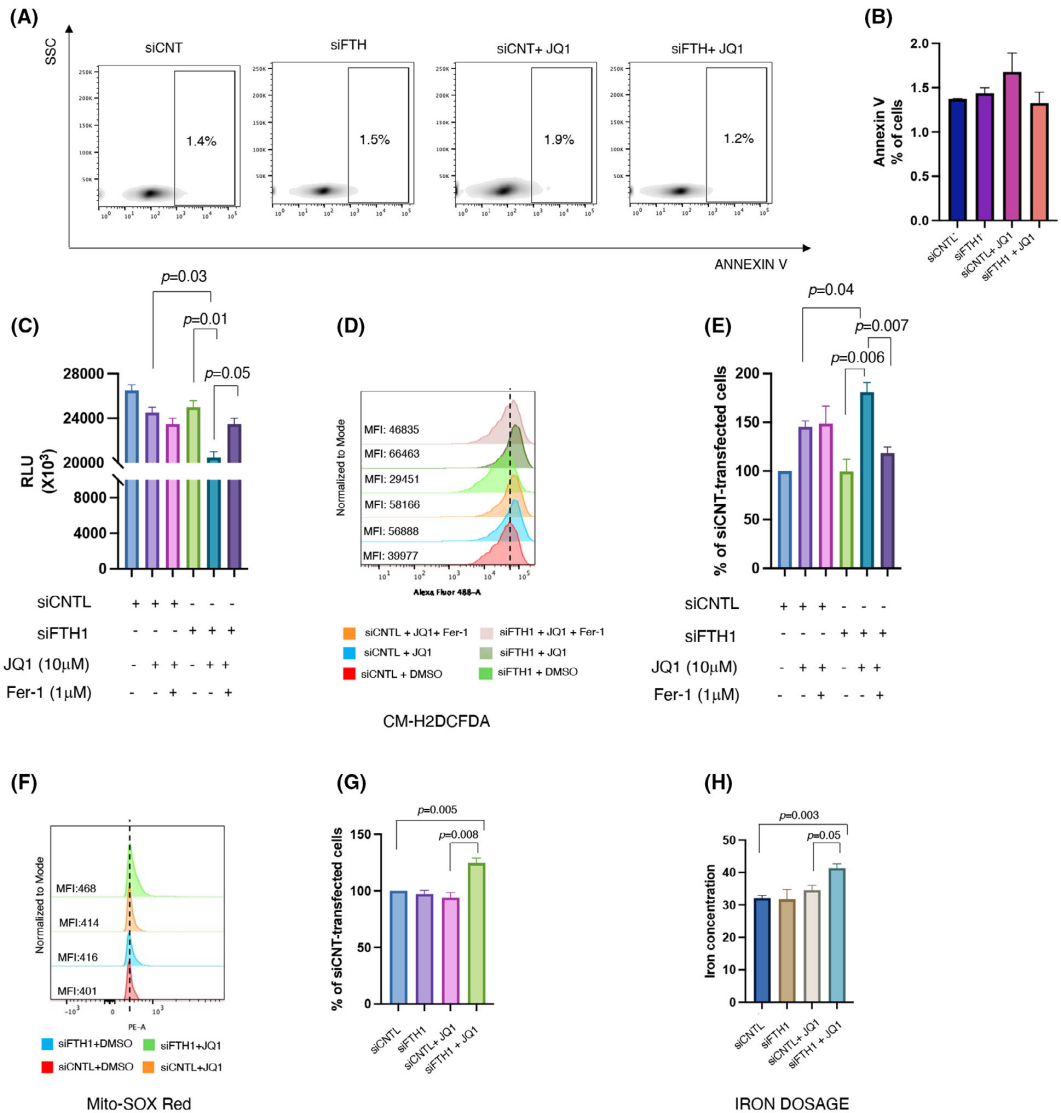
*FTH1* silencing combined with BET inhibitor JQ1 leads to the anticancer effect, inducing ferroptosis in NCI‐H460 JQ1‐insensitive cells. (A) Representative density plot of Annexin V binding assay per side scatter (SSC; granularity)of NCI‐H460^siCNTL^ and NCI‐H460^siFTH1^ cells analyzed by flow cytometry. (B) Bar diagram of the percentage of apoptotic cells (Annexin V‐positive cells) as measured by Annexin V binding assay. Values (mean ± SEM; *n* = 3) are shown. A statistically significant difference was determined by Student's *t* test. The absence of *P* values in the bar diagram indicates that there are not statistically significant differences. (C) Cells (3 × 10^6^) were transiently transfected with a pool of siFTH1or siRNA control. Twenty‐four hours later, transfected cells were treated or not with JQ1, and treated or not with Fer‐1 (1 μm). Cells were lysed 24 h later. The luminescence in relative light units (RLU) was plotted. Viability was determined in technical triplicate by CellTiter‐Glo assay. We use the symbols ‘+’ or ‘−’ to indicate the presence or the absence of JQ1, respectively. We use the symbols ‘+’ or ‘−’ to indicate the presence or the absence of Fer‐1, respectively. We use the symbols ‘+’ or ‘−’ to indicate the presence or the absence of each vector, respectively. We use the symbols ‘+’ or ‘−’ to indicate the presence or the absence of each vector, respectively. Data shown are representative results of three independent experiments. Values (mean ± SEM; *n* = 3) are shown. A statistically significant difference according to Student's *t* test. (D) Flow cytometry histogram showing the analysis of cytosolic ROS (Reactive oxygen species) measured upon staining with CM‐H2DCFDA in NCI‐H460 cells transiently transfected with a pool of siFTH1 or siRNA control, treated or not with JQ1, and treated or not with Fer‐1 (Ferrostatin‐1) 1 μm. The vertical dashed line indicates the cut‐off relative to the control cells (siCNTL + DMSO). (E) Bar diagram showing ROS levels presented as the % of control (siCNTL transfected cells). We use the symbols ‘+’ or ‘−’ to indicate the presence or the absence of JQ1, respectively. We use the symbols ‘+’ or ‘−’ to indicate the presence or the absence of Fer‐1, respectively. We use the symbols ‘+’ or ‘−’ to indicate the presence or the absence of each vector, respectively. We use the symbols ‘+’ or ‘−’ to indicate the presence or the absence of each vector, respectively. (F) Flow cytometry histogram showing the analysis of mitochondrial ROS (mitoROS) quantified by using MitoSOX Red of NCI‐H460 cells transiently transfected with a pool of siFTH1 or siRNA control and treated or not with JQ1. (G) Bar diagram showing mitoROS levels presented as the % of control (siCNTL transfected cells). The vertical dashed line indicates the cut‐off relative to the control cells (siCNTL + DMSO). (H) Bar diagram showing the intracellular ferrous iron levels determined using iron colorimetric assay in NCI‐H460 cells transiently transfected with a pool of siFTH1 or siRNA control and treated or not with JQ1. Data were statistically analyzed by Student's *t* test and are reported as mean values ± SEM of three independent experiments.

It has been reported that JQ1 might induce cell death by either triggering apoptosis or ferroptosis [41, 49, 50, 51, 52]. To dissect the type of regulated cell death (RCD) promoted by *FTH1* silencing in NCI‐H460 cells upon JQ1 treatment, we first performed an Annexin V flow cytometric assay and found that the co‐treatment did not induce apoptotic cell death in H460 cells (Fig. 6A,B). Hence, we applied ferrostatin‐1 (Fer‐1), an inhibitor of ferroptosis, to explore if ferroptosis is the type of cell death induced by JQ1. By measuring cell viability (Fig. 6C), we confirmed that the combined use of JQ1 with *FTH1* silencing triggered cell death compared to JQ1 alone (*P* = 0.03) or NCI‐H460^siFTH1^ cells (*P* = 0.01) (as showed in Fig. S3C). Notably, in cells treated with Fer‐1, JQ1/*FTH1* silencing‐induced cell death was noticeably inhibited (*P* = 0.05) (Fig. 6C). Furthermore, we performed an intracellular total ROS detection assay in NCI‐H460 cells treated with JQ1 alone or in combination with *FTH1* silencing with or without Fer‐1 (Fig. 6D,E). We observed that JQ1 treatment alone and in combination with *FTH1* silencing increased the intracellular content of total ROS (Fig. 6D,E). The treatment with Fer‐1 let uncharged the intracellular total ROS content upon JQ1 treatment, while reversed the increase of intracellular total ROS content due to JQ1/*FTH1* silencing (Fig. 6D,E).

We proceed to investigate the other main hallmarks of this type of cell death, namely mitochondrial ROS, labile iron pool (LIP), and lipid peroxidation in NCI‐H460 cells treated with JQ1 alone or in combination with *FTH1* silencing. We found that JQ1 treatment alone did not alter the mitochondrial ROS (Fig. 6F,G). The combination of JQ1 with *FTH1* silencing, instead, induced a significant increase in mitochondrial ROS content (Fig. 6F,G). Moreover, as shown in Fig. 6 Panel H, the LIP level was increased only in the cells treated with the combination of JQ1 and *FTH1* depletion. Strikingly, lipid peroxidation measured by C11‐BODIPY 581/591 assay did not significantly increase either in the NCI‐H460 cells treated with JQ1 alone or in cells treated with the combination of JQ1 and FTH1 depletion (Fig. S6A,B).

**Fig. 7:**
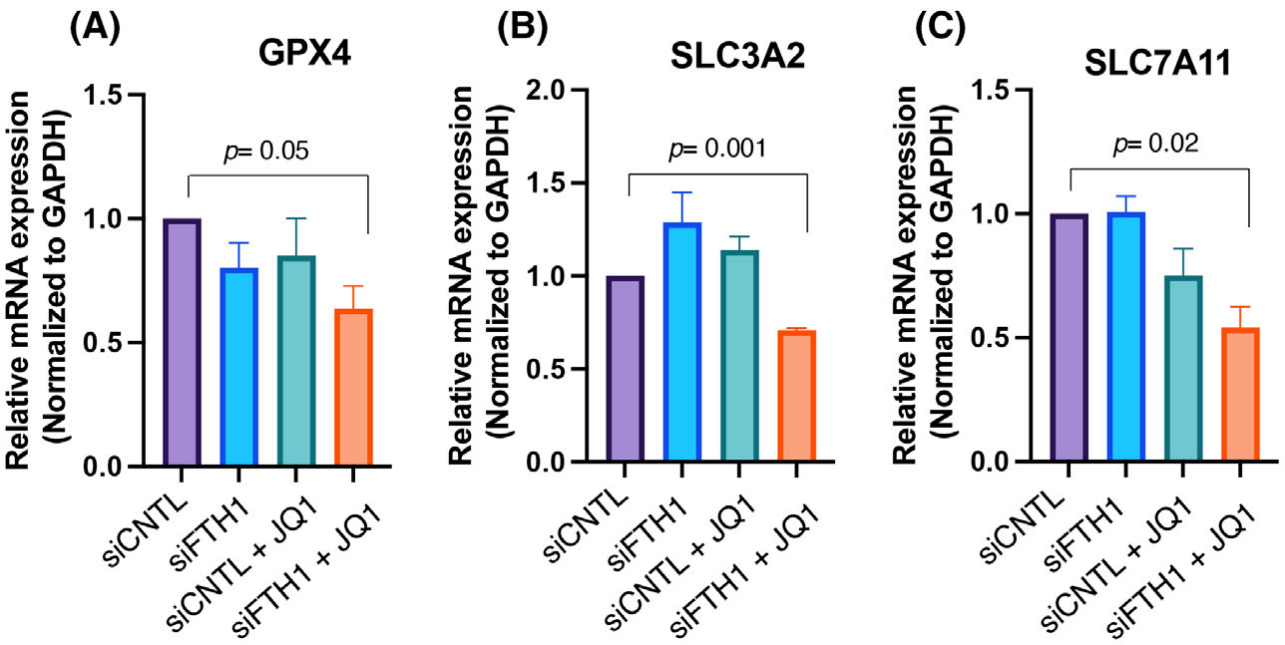
*FTH1* silencing combined with BET inhibitor JQ1 impaired mRNA expression of ferroptosis‐associated genes in NCI‐H460 JQ1‐insensitive cells. (A–C) Total RNA was extracted from NCI‐ H460^siCNTL^ and NCI‐ H460^siFTH1^ cells w/o JQ1 treatment and analyzed by real‐time PCR for the expression of *GPX4*, *SLC3A2*, and *SLC7A11*, respectively. Results were normalized using *GAPDH* as the housekeeping gene. Data were statistically analyzed by Student's *t* test and are reported as mean values ± SEM of three independent experiments.

Because BRD2 has been reported as a super enhancer for gene transcription [53, 54], we investigated whether inhibition of BRD2 via JQ1 treatment with or without *FTH1* silencing could regulate the transcription of ferroptosis‐associated genes *GPX4*, a ROS scavenger, and the expression of solute carrier family 7 member 11 (*SLC7A11*) and solute carrier family 3 member 2 (*SLC3A2*), which inhibit ferroptosis via the production of reduced glutathione [55]. The results showed that JQ1 treatment alone does not alter the expression of *GPX4*, *SLC7A11*, and *SLC3A2* compared to untreated cells (Fig. 7A–C). The results, shown in Fig. 7, indicate that the association of *FTH1* silencing with JQ1 treatment is the only one able to significantly downregulate the steady‐state amounts of *GPX4*, *SLC7A11*, and *SLC3A2*.

## Discussion

Resistance is the main cause of therapeutic failure in non‐small cell lung cancer (NSCLC) even with the use of advanced therapies such as immunotherapies, radiotherapies, and chemo‐immunotherapies, leading to cancer recurrence and metastasis, and reducing patient lifespans [56]. To improve the overall survival rate of lung cancer patients, understanding the molecular mechanism that can help overcome resistance is crucial. Evidence suggests that the link between intracellular iron metabolism and lung tumorigenesis is well established [57, 58].

An intriguing feature of the ferritin‐heavy subunit is represented by its ability to physically interact with other proteins and, in some well‐established cases, modulate their activity. The list of FTH1 interactors is continually growing and includes the chemokine receptor CXCR4, whose activity is negatively modulated upon the interaction [40], the tumor suppressor p53, of which the transcriptional activity is incremented [39], the DNA replication regulatory protein NCOA4 that targets FTH1 for degradation [59], the Alacrima–Acalasia–Adrenal Insufficiency Neurological Disorder (ALADIN) protein involved in the triple A syndrome [60], and the Death Domain‐Associated nuclear protein (DAXX) [61], together with FTH1 can participate in apoptotic pathways. In recent years, the membrane‐bound protein associated with metabolism and energy FAME [62] and the antioxidant enzyme PRDX6 [31] have been added to this list.

It has also recently been demonstrated that Alpha B crystallin (CRYAB), a molecular chaperone, binds to FTH1 and stabilizes it, inhibiting its proteasomal degradation [63]. Furthermore, FTH1 was identified as a direct target of a Methyltransferase‐like 14 (METTL14) which reduces its expression through the degradation of mRNA [64].

The binding of FTH1 with molecules involved in different metabolic pathways suggests that some of these interacting molecules might themselves be involved in iron metabolism, as has been recently proposed for FAME [62]. Conversely, FTH1, in addition to its fundamental role in iron metabolism, could perform critical functions also in metabolic pathways not strictly related to iron management. Indeed, others and our works in the last years identified FTH1, defined as a moonlighting protein, as a multifunctional protein involved in multiple metabolic routes from the control of cell proliferation to angiogenesis [65], immunomodulation [66], expression of specific sets of oncomiRNAs [67], induction of epithelial to mesenchymal transition [68], and propagation of cancer stem cells [69].

BET proteins are well known for their ability to recruit transcriptional co‐regulators to acetylated chromatin. Even though the function of the BRD2 protein is not fully understood, it is being actively investigated as a therapeutic target for multiple diseases.

In this study, we have attempted to evaluate the impact of BRD2‐FTH1 interaction in lung cancer cells to understand the functional role of this interaction in NSCLC progression. We expanded our understanding of the mechanistic function of FTH1, emphasizing how its depletion can act on the regulation of ferroptosis, a promising way to overcome drug resistance and enhance the therapeutic efficacy of anticancer treatment. We also aimed to determine whether the FTH1/BRD2 interaction could be involved in this process.

We found that FTH1 physically interacts with BRD2. The BRD2‐FTH1 complex is situated within the HEK293T nuclei, as confirmed by immunofluorescence analysis. Noteworthy, we showed the first case of nuclear BRD2‐FTH1 interaction. We reasoned on which domain of FTH1 could be involved in the BRD2 binding and, in turn, the BRD2 domains involved in the FTH1 interaction. To the best of our knowledge, previous studies on FTH1 interactions have not yet highlighted a functional domain involved in these interactions [31, 70]. On the BRD2 protein, unlike other BET proteins, recent studies identified a region rich in Ser, Asp, and Glu residues denominated SEED domain within the extra‐terminal domain (ET) at the C terminus, and an exclusive acidic region (Ac) before the dimerization domain, named motif B (mD), involved in specific protein interaction [71, 72]. Ongoing studies are focusing on characterizing the functional domains of both FTH1 and BRD2 proteins involved in this interaction.

We proceed with investigating the expression of BRD2 in data from CPTAC, a publicly available resource that provides protein expression and clinical information of thousands of cancer patients [73]. Our observation in a dataset from 111 lung adenocarcinoma (LUAD) patients indicates that levels of BRD2 positively correlate with different stages of cancers. For this reason, we demonstrated the nuclear localization of FTH1 and the nuclear co‐localization of BRD2 and FTH1 in a panel of NSCLC *in vitro*. Intriguingly, the immunofluorescence analysis reveals a distinct difference in co‐localization intensity among the three cell lines investigated. Unlike BRD2, FTH1 did not co‐localize with the most well‐studied BET family member BRD4. In addition, *FTH1* silencing decreases the BRD2 protein level without modifying the BRD2 gene expression. These results suggest that FTH1 exclusively acts at the post‐transcriptional level of BRD2 regulation. The mechanism underlying this decrease in BRD2 expression is unknown; however, we theorized that FTH1 could be involved in the regulation of BRD2 stability through mechanisms leading to protein degradation, as shown by MG132 treatment upon *FTH1* silencing, either autophagy‐mediated or ubiquitin signaling mediated. In our opinion, it significantly enhances our understanding of molecular mechanisms underlying FTH1‐dependent BRD2 protein regulation.

Previous findings showed the ability of nuclear FTH1 to protect the DNA of corneal epithelial cells from UV damage and oxidative stress [5, 6, 74]. The downregulation of *FTH1* expression in cancer cells both *in vitro* and *in vivo* resulted in increased vulnerability of glioma U251, sNF96.2, and breast cancer MCF‐7 cells to chemotherapy [4, 10]; based on these reports, we hypothesized that downregulation of FTH1 in NSCLC cells could provide a mechanism to increase the chemotherapeutic sensitivity of lung cancer by eliminating the protective functions of FTH1. To test our hypothesis, we verified the vulnerability of *FTH1‐silenced* tumor cells to JQ1, a pan‐BET inhibitor, initially identified as a BRD4 inhibitor, and then, it has been shown to inhibit multiple BET protein members due to the well‐preserved bromodomain sequence [14, 75].

Our data showed significant results on NSCLC (*KRAS/LKB1* mutant) insensitive rather than NSCLC (*KRAS* mutant) sensitive cells to JQ1 treatment, identifying the combination of JQ1 and *FTH1* depletion as a potential therapeutic option for the treatment of the more aggressive NSCLC insensitive cells, enhancing the JQ1 anti‐tumor effects.

In addition, we demonstrated a correlation between the combination of JQ1/*FTH1* depletion and ferroptosis. Few recent reports correlate JQ1 and ferroptosis [51, 52]. Sui *et al*. [52] showed that JQ1 induced ferroptosis via ferritinophagy in cancer cells, targeting BRD4. However, recent observations correlate *LKB1* depletion to ferroptosis by increasing lipid peroxidation [76]. Intriguingly, Zhang *et al*. [77] reported a novel FTH1‐dependent mechanism that affected ferroptosis sensitivity in LUAD cells due to an increase in labile iron. For the first time, it was observed that the glutamate accumulation suppressed Yes‐associated protein (YAP) through the ADCY/PKA/HBP/YAP axis, leading to the inability to sustain the transcriptional compensatory of FTH1 caused by ferritinophagy [77]. Moreover, even more recent findings demonstrated a correlation between the LKB1/AMPK pathway and impaired NRF2 activity triggering ferroptosis in a neurodegenerative disease [78]. Probably, the ferroptotic cellular response to the combination of JQ1 and FTH1 depletion, as shown for the first time in the current study, could have some association with *LKB1* gene status. Surely, deeper findings are needed to investigate the signaling network of *LKB1*.

Of note, we found that the expression levels of ferroptosis‐associated genes *GPX4*, *SLC7A11*, and *SLC3A2* were decreased under the combined treatment with JQ1/*FTH1* depletion. Because BRD2 is a transcriptional regulator [79, 80, 81], we hypothesized that the decreased levels of BRD2 due to *FTH1* silencing contributed to the downregulation of ferroptosis‐associated genes *GPX4*, *SLC7A11*, and *SLC3A2* mediated by JQ1 as described previously [52]. However, the specific mechanisms by which BRD2 regulates the expression of the ferroptosis‐associated genes need further investigation.

In the current study, we also demonstrated that the combination treatment of JQ1/*FTH1* silencing led to an increase in: (a) LIP, (b) intracellular, and (c) mitochondrial ROS. Strikingly, although lipid peroxidation is considered a hallmark of ferroptosis [82], no increase of intracellular lipid hydroperoxides has been observed. The impaired expression of *GPX4*, *SLC3A2*, and *SLC7A11* after the combination of *FHT1* silencing and JQ1 treatment seemed not to be sufficient to induce lipid peroxidation on its own. For the moment, the molecular basis that underlies this non‐canonical pathway is not fully understood; however, recent studies also highlighted that FINs such as Erastin may promote ferroptosis in gastric, lung, and ovarian cancer without impairing lipid peroxidation [83, 84, 85]. Future focused studies are necessary to provide mechanistic insights into this odd.

**Fig. 8:**
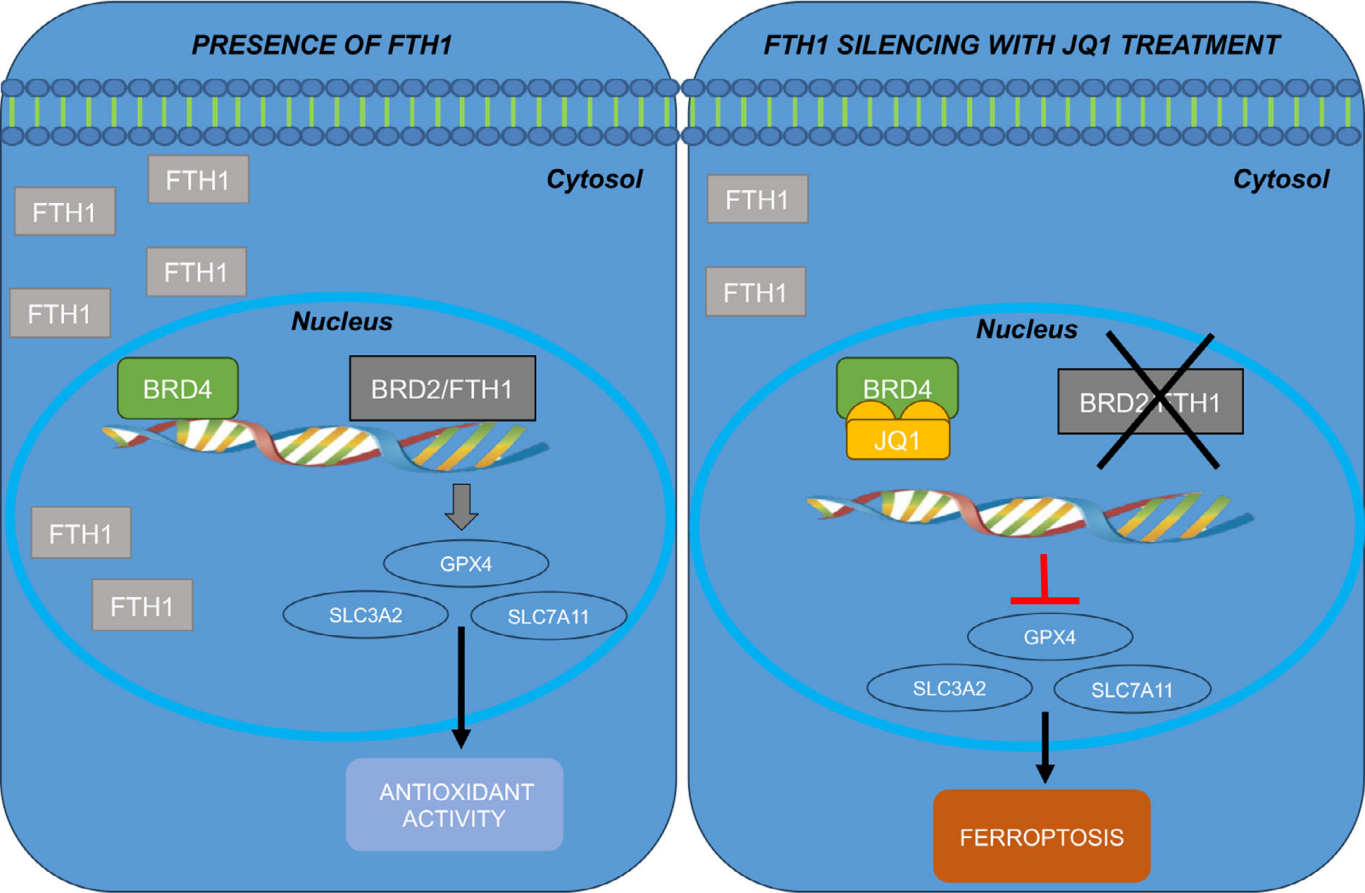
Schematic diagram of the co‐treatment of JQ1/*FTH1* silencing. In basal condition (left panel), FTH1 is bound with BRD2 in the nuclei of JQ1‐insensitive NSCLC cells and improves DNA protection from oxidative damage. *FTH1* silencing combined with BET inhibitor JQ1 treatment (right panel), leads to the inhibition of cell growth, which mainly resulted from the upregulation of ferroptosis in cells.

In summary, our model confirms the protective role of FTH1 in the nucleus from oxidative stress, through transcriptional regulation mediated by BRD2. The protective role of nuclear FTH1 in cancer cells demonstrates that reducing FTH1 levels, increases the drug sensitivity to JQ1, inducing cell death through ferroptosis (Fig. 8 Schematic diagram).

The ferritin inhibitors are not yet widely used in clinical anti‐tumor therapy. However, there are some ongoing research studies exploring their potential therapeutic use in cancer treatment. Deferoxamine (DFO) [86, 87] and Deferasirox (DFX) [87, 88], iron chelating agents, have been used in some clinical studies to treat iron overload (siderosis) in cancer patients, but not as a specific anti‐tumor treatment. Cellular pathways that influence ferritin levels, such as ferritinophagy and ferroptosis, may be targetable for therapeutic purposes. Of note, a study by Yang *et al*. reported that artesunate, an anti‐malaria drug used for cancer treatment, caused ferroptosis in HeLa and hepatocellular carcinoma, HepG2 cells, making it the first report of FTH1 involvement in ferroptosis. This study demonstrated that *FTH1* overexpression directly and indirectly was capable of reversing artesunate‐mediated ferroptosis [89, 90]. Moreover, Sui *et al*. [52] demonstrated that JQ1 enhanced ferroptosis via the increase in ferritinophagy. In this process, FTH1 is degraded via autophagy, leading to increased intracellular levels of iron.

Briefly, while ferritin inhibitors are not yet widely used in clinical anti‐tumor therapy, there are ongoing research studies exploring their potential therapeutic use in cancer treatment alone or in combination with chemotherapeutics to treat more aggressive and drug‐resistant tumors.

This report shows a novel co‐targeting strategy by combined inhibition of BRD2 through BET inhibitor JQ1 and *FTH1*depletion in unresponsive non‐small cell lung cancer cells (NSCLC) to JQ1. Our data suggest that this combined treatment represents a potential therapeutic approach in more aggressive NSCLC cell types and highlights the importance of BRD2‐FTH1 interaction in lung tumorigenesis and development by inducing ferroptosis.

## Materials and methods

### Cell culture, transient expression, and treatments

NSCLC cell lines NCI‐H23 (RRID:CVCL_1547), NCI‐H460 (RRID:CVCL_0459), and A549 (RRID:CVCL_0023) cells from ATCC were grown in RPMI (Thermo Fisher Scientific, Waltham, MA, USA). Of note, NSCLC cell lines (NCI‐H23, NCI‐H460, and A549 cells) have different origins. NCI‐H23 and A549 cells were isolated from the lung tissue of primary lung adenocarcinoma; NCI‐H460 cells derived from the metastatic pleural fluid of a patient with large cell lung cancer [91]. About the genetic profile, all three NSCLC cell lines harbor an activating oncogenic mutation in the GTPase domain of the *KRAS* signaling protein [35, 38]. NCI‐H460 and A549 cells co‐occur genetic alteration of *KRAS* and the Liver kinase B1 (*LKB1*) tumor suppressor gene [36, 38, 41]. The mutation in *LKB1* results in LKB1 protein loss [36]. HEK293T cells (ATCC; RRID:CVCL_0063) were grown in Dulbecco's modified Eagle medium (DMEM; Thermo Fisher Scientific, Waltham, MA, USA). Cell culture media were supplemented with 10% fetal bovine serum (FBS), 100 U·mL^−1^ penicillin, and 100 μg·mL^−1^ streptomycin; all reagents were purchased from Thermo Fisher Scientific. All of the media, sera, and reagents used in experiments are obtained from mycoplasma‐free sources. All experiments were performed with mycoplasma‐free cells.

The plasmids pCMV6‐FTH1‐FLAG and pCMV6 were from OriGene Technologies, Inc. (Rockville, MD, USA). JQ1(BK84566S_CST) was purchased from Cell Signaling Technology, Inc. (Danvers, MA, USA). For the downregulation of FTH1 and BRD2 expression, a pool of small interfering RNAs (siRNAs) targeting FTH1 and BRD2 was purchased from Dharmacon (Lafayette, CO, USA). For JQ1 treatments, NCI‐H23 and NCI‐H460 cells were plated in a 6‐well plate in a complete medium. The next day, cells were transiently transfected with 434 pmol siRNA or the corresponding controls using Lipofectamine 3000 (L3000001, Thermo Fisher Scientific) according to the manufacturer's protocol. After 24 h, cells were treated with JQ1 at 10 μm for 24 h. The used JQ1 concentration and time of exposure agreed with previous literature studies [28, 52].

### Immunofluorescence

Cells were grown in Nunc Lab‐Tek chamber slides, washed in PBS, fixed with 4% freshly made formaldehyde for 10 min at room temperature (RT). Cells were permeabilized with 0.5% Triton X‐100 in PBS for 10 min at RT and blocked with 1% BSA in 0.1% Triton X‐100 in PBS for 30 min. Primary antibodies were diluted in blocking buffer and applied to cells overnight in the cold room with gentle rotation [92]. Primary antibodies used are anti‐FTH1 (Santa Cruz Biotechnology, Dallas, TX, USA, sc‐376594), anti‐BRD2 (Cell signaling Technology, #5848S), anti‐BRD4 (Thermo Fisher Scientific, PA5‐100998). After three washes, secondary antibody (anti‐mouse IgG Alexa Fluor 488 and anti‐rabbit IgG Alexa Fluor 633, Thermo‐Fisher Scientific) conjugated with appropriate fluorophores were applied to the cells and incubated at 37 °C for 1 h. After three washes, DAPI (Sigma Aldrich, St. Louis, MO, USA) was applied. Immunofluorescence was performed under the Leica THUNDER Imaging Systems DMi8 for FTH1‐BRD4 co‐localization using a ×63 oil objective, and Leica Stellaris 5 Confocal Microscope (Leica Microsystems S.r.l., Wetzlar, Germany) for FTH1‐BRD2 co‐localization using a ×63 oil objective. Single images were captured with a z size of 7–7.5 μm and for a z series, a 0.38 μm step interval was used.

### Quantitative analysis of localization and co‐localization studies

Quantification of FTH1 nuclear and cytoplasmic localization intensity, and BRD2‐4/FTH1 co‐localization intensity was determined in NHI‐H23, NHI‐H460, and A549 cell lines.

Quantification of FTH1 as Mean Fluorescence Intensity (MFI) per cell was performed using fiji‐imagej processing free software (http://rsb.info.nih.gov/ij/).

We measured the fluorescence of FTH1 in the selected z‐stack of 8‐bit images of the nucleus and cytoplasm per cell. In both cases, we obtained the mean intensity values (fluorescence intensity/nuclear‐cytoplasmic area) provided in arbitrary units by the fiji tool on 63× images. All cell nuclei were delineated using the freehand selections tool of fiji. The delineated nuclei represent our ROIs (Regions of interest). All cell cytoplasmic areas were delineated by excluding the nuclear areas from the entire cells. The delineated cytoplasmic areas represent our ROIs.

The degree of co‐localization was calculated using fiji‐imagej processing free software, BIOP‐JACOP plugin. The manual threshold for the fluorescence intensity of each channel was carefully adjusted for background determination and was followed by Manders' Overlap coefficient (MOC) calculation on ROIs selected (represented by nuclei and cytoplasmic regions). Manders' overlap coefficient (MOC) expresses the proportion of each signal (red) that overlaps with the other (green) and has been designed according to the equation:

$$\text{Overlap}=\frac{\sum_{i}A_{i}\times B_{i}}{\sqrt{\sum_{i}{\left(A_{i}\right)}^{2}\times \sum_{i}{\left(B_{i}\right)}^{2}}}$$

where *A*_*i*_ and *B*_*i*_ represent intensities of the pixel *i* on images A (red) and B (green), respectively. This results in a numerical value in the 0–100% range, where a coefficient value over 50% corresponds to co‐localization [93, 94].

For each cell line, the average for MOC percentages was calculated. Image manipulation was performed with Photoshop CS2 (Adobe, Chatswood, Australia).

### Public data access and analysis

A dataset retrieved by The Clinical Proteomic Tumor Analysis Consortium (CPTAC) was used to explore the expression profile and clinicopathological characteristics of the BDR2 protein in Lung Adenocarcinoma (LUAD). Results were presented as a box plot. *Z*‐values represented standard deviations (SD) from the median across samples for the lung adenocarcinoma (LUAD) cancer type. Log_2_ spectral count ratio values from CPTAC were first normalized within each sample profile and then normalized across samples. The protein expression of BDR2 in the CPTAC database was assessed by ULCAN (http://ualcan.path.uab.edu/analysis.html) (accessed on 1 August 2024).

### Cell viability, cell death, and apoptosis assay

The growth rates of NCI‐H460 and NCI‐H23 were determined using a trypan blue dye exclusion assay. Cells were transiently transfected with a pool of siBRD2 or siFTH1 or the corresponding controls. After 24 h, cells were treated or not with JQ1. Cell viability of NCI‐H460 and NCI‐H23 was also evaluated with the CellTiter‐Glo® Luminescent Cell Viability Assay (Promega, Madison, WI, USA), which is based on quantitation of ATP as an indicator of metabolically active cells [95, 96]. A total of 3.0 × 10^3^ cells per well were plated in 96 wells overnight. The next day, cells were transiently transfected with a pool of siBRD2 or siFTH1 or the corresponding controls. After 24 h, cells were treated or not with JQ1. An Annexin V‐based apoptotic assay was performed as previously described [97]. Briefly, NCI‐H460 cells (1.0 × 10^6^) were stained with FITC‐conjugated Annexin V using the Annexin V‐FITC kit (Miltenyi Biotech, Cologne, Germany). Data were collected by flow cytometry. A cell death assay was performed as previously described [98]. Briefly, NCI‐H460 and NCI‐H23 cell lines, 5.0 × 10^5^ cells were seeded in 6‐well plates overnight. The next day, cells were transiently transfected with a pool of siBRD2 or siFTH1 or the corresponding controls. After 24 h, cells were treated or not with JQ1. Cells were incubated with PI solution in the dark at 37 °C for 15 min. Samples were then washed twice with PBS. A total of 2.0 × 10^4^ events were acquired by a FACS BD LSRFortessa™ X‐20 cytofluorometer (BD Biosciences, Milan, Italy). PI‐positive cells were analyzed by the flowjo software program (Tree Star, Inc., Ashland, OR, USA).

### Measurement of intracellular and mitochondrial ROS

Intracellular and mitochondrial ROS analyses were performed as previously described [98, 99]. Briefly, the levels of intracellular ROS were determined by incubating cells for 10' at 37 °C with the redox‐sensitive probe 2'‐7'‐Dichlorodihydrofluorescein diacetate (CM‐H2DCFDA; Thermo Fisher Scientific) according to the manufacturer's instructions. CM‐H2DCFDA fluorescence was collected by flow cytometry, and data were analyzed with flowjo software (Tree Star, Inc.). For mitochondrial ROS analyses, cells were incubated with 5 μm MitoSOX Red (MitoSOX Red Mitochondrial Superoxide Indicator, Thermo Fisher Scientific) for 10 min at 37 °C, and then, 2.0 × 10^4^ cells were acquired by flow cytometry. Fluorescence data were processed with flowjo software (Tree Star, Inc.).

### Measurement of the labile iron Pool (LIP) level

Intracellular ferrous iron levels were determined using an Iron Colorimetric Assay Kit purchased from Abcam (ab83366; Cambridge, UK). According to the manufacturer's instructions, cells were added to iron assay buffer, homogenized on ice, and centrifuged at 16 000 ***g*** for 10 min at 4 °C to obtain the supernatant for the assay. A 50‐μL sample was incubated in a 96‐well microplate for 30 min at 37 °C. Then, the sample was incubated with 100 μL of reagent mix in the dark for 60 min at 37 °C, and the absorbance was measured at 593 nm with a microplate reader.

### BODIPY™ assay

Lipid peroxidation was investigated by flow cytometry using BODIPY™ 581/591 C11 dye (Thermo Fisher Scientific), as previously described [99]. Briefly, cells were seeded in 6‐well plates at a density of 1.5 × 10^5^ cells per well and grown overnight. After treatments, cells were loaded with 2.5 mm BODIPY™ 581/591 C11 for 30 min at 37 °C. After 30 min of loading, unincorporated dye was removed by washing twice with PBS. Samples were then centrifuged at  200 ***g*** for 5 min, and the pellets were resuspended in 300 mL of PBS. The cell suspension was subjected to the flow cytometry analysis to analyze the amount of lipid ROS within cells. Oxidation of BODIPY‐C11 resulted in a shift of the fluorescence emission peak from ~ 590 to ~ 510 nm proportional to lipid ROS generation [100].

The fluorescence intensities of cells per sample were determined by flow cytometry using the FACS BD LSRFortessa™ X‐20 cytofluorometer (BD Biosciences). A minimum of 20 000 cells were analyzed per condition. Fluorescence of each probe was measured using flowjo software program (Tree Star, Inc.). Each experiment was performed in triplicate.

### Quantitative real‐time PCR

Total RNA was isolated from cells using PureLink RNA Mini Kit (Thermo Fisher Scientific). After DNase treatment, cDNA was synthesized by Applied Biosystems™ High‐Capacity cDNA Reverse Transcription Kit (Thermo Fisher Scientific), according to the manufacturer's instructions. Real‐time PCR was performed with the SYBR™ Green qPCR Master Mix (Thermo Fisher Scientific) using a quant studio 3 instrument (Thermo Fisher Scientific) [101]. Real‐time PCR results were analyzed using quant studio real‐time pcr Software (Thermo Fisher Scientific). Reactions were carried out in triplicate, and gene expression levels were calculated relatively to GAPDH mRNA levels as endogenous control. Real‐time PCR amplification values were reported as the $2^{-\Delta \Delta C_{t}}$ method [102]. The following primers were used: human BRD2 gene [103], 5′‐gtcaaactgggtctaccggatt‐3′ and 3′‐cttttccagcgtttgtgcca‐5′; human FTH1 gene, 5′‐ttgaccgagatgatgtggct‐3′ and 3′‐ccagtttgtgcagttccagt‐5′; human GAPDH gene, 5′‐caaattccatggcaccgtca‐3′ and 3′‐ggcagagatgatgacccttt‐5′; human BRD4 gene [103], 5′‐acaacaagcctggagatgaca‐3′ and 3′‐gtttggtaccgtggaaacgc‐5′; human GPX4 gene, 5′‐atcgacgggcacatggttaa‐3′ and 3′‐cgacgagctgagtgtagttt‐5′; human SLC3A2 gene, 5′‐caactaccggggtgagaact‐3′ and 3′‐ttgccactcagccaagaact−5′; human SLC7A11, 5′‐gcaacaaagatcggaactgct‐3′ and 3′‐gctggctggttttacctcaac‐5′.

### Immunoprecipitation

Immunoprecipitation (IP) experiments were performed as previously described [31]. Briefly, lysates from 3xFlag‐FTH1 transfected 293T HEK cells and 3xFlag negative control (cells transfected with empty vector) were pre‐cleared onto DynabeadsTM Protein G (Invitrogen, Waltham, MA, USA); cellular extracts were subjected to overnight immunoprecipitation using anti‐FLAG M2 (Sigma Aldrich) magnetic beads. The 5% of the lysate was kept at 4 °C as input. Supernatants containing the unbound proteins were removed, and the beads were washed with two different NaCl concentrations (150 and 300 mm) in lysis buffer. The beads were collected by centrifugation and denatured for 10 min at 70 °C in 25 mL of 2× Nupage sample buffer (Life Technologies, CA, USA). Protein samples were loaded onto 4–12% NuPAGE Novex Bis‐Tris protein gradient polyacrylamide gels (Thermo Fisher Scientific).

### Western blotting

Total extracts were prepared as previously described [104]. This method has also been described in earlier papers [95, 98]. Briefly, to obtain total protein extracts, cells were washed once with PBS (1×) and total cell lysates were prepared using RIPA [95]. The samples were centrifuged at 13800 ***g*** for 20 min at +4 °C, and supernatants containing the total extracts were recovered. Proteins were separated on 4–12% NuPAGE Novex Bis‐Tris protein gradient polyacrylamide gels (Thermo Fisher Scientific) and blotted onto nitrocellulose. Membranes were blocked with 5% milk (BioRad, CA, USA) and then incubated with the following antibodies: anti‐FTH1 (Cell Signaling #4393S), anti‐GAPDH‐HRP‐conjugated (Santa‐Cruz, sc‐47724), anti‐BRD2 (Cell Signaling #5848S), and anti‐BRD4 (Invitrogen, PA5‐100998). Peroxidase AffiniPure Sheep Anti‐Mouse IgG, Peroxidase AffiniPure Donkey Anti‐Rabbit IgG, and Peroxidase AffiniPure Donkey Anti‐Goat IgG (1 : 10 000, Jackson ImmunoResearch Europe Ltd. Cambridge House) secondary antibodies were used. Signals were detected using the WESTARï2.0 ECL substrates for western blotting (XLS070,0250, Cyanagen, Bologna, Italy) and acquired using a Uvitec Alliance Mini HD9 (Uvitec, Cambridge, UK).

### Statistical analysis

Statistical analysis was performed by the two‐tailed unpaired Student's *t* test using graphpad prism® software package (GraphPad Software, Inc., CA, USA). Statistical significance was determined by *P* < 0.05.

## Conflict of interest

The authors declare no conflict of interest.

## Author contributions

SS and CG contributed to performing experiments and analyzing data. BS assisted with the experiments. GS performed computational analysis. FC, MM, and VM helped to analyze the data. FB helped to interpret data and review the manuscript. EV conceived, designed, investigated, edited the original draft, and supervised the study. MCF coordinated the project and reviewed the manuscript.

## Supporting information


**Fig. S1.** Ferritin heavy chain does not interact with BRD4 in a panel of NSCLC cells.
**Fig. S2.** Cell viability analysis by Cell titer Glo in NCI‐H23, and NCI‐H460 cell lines 24 h after treatment with JQ1 at the indicated concentrations.
**Fig. S3.** Ferritin heavy chain is not involved in BRD2 mRNA alteration in NSCLC cells.
**Fig. S4.** *FTH1* silencing combined with BET inhibitor JQ1 inhibits the growth of NCI‐H460 JQ1‐insensitive cells. The same effect is observed when BRD2 is knocked down.
**Fig. S5.** Ferritin heavy chain does not influence BRD4 levels in NCI‐H460 cells.
**Fig. S6.** Analysis of lipid peroxidation by flow cytometry.

## Data Availability

Data are available from the corresponding author upon reasonable request.
