# Supplementary material for: Overcoming BET‐inhibitor JQ1 resistance in aggressive non‐small cell lung cancer by inducing ferroptosis via inhibition of the BRD2–FTH1 axis
Source: FEBS J. 2025 Jul 13;292(23):6345–64. doi: 10.1111/febs.70191 (PMC12699187; doi:10.1111/febs.70191)
Supplement: Supplementary file 1 — Fig. S1. Ferritin heavy chain does not interact with BRD4 in a panel of NSCLC cells. Fig. S2. Cell viability analysis by Cell titer Glo in NCI‐H23, and NCI‐H460 cell lines 24 h after treatment with JQ1 at the indicated concentrations. Fig. S3. Ferritin heavy chain is not involved in BRD2 mRNA alteration in NSCLC cells. Fig. S4. FTH1 silencing combined with BET inhibitor JQ1 inhibits the growth of NCI‐H460 JQ1‐insensitive cells. The same effect is observed when BRD2 is knocked down. Fig. S5. Ferritin heavy chain does not influence BRD4 levels in NCI‐H460 cells. Fig. S6. Analysis of lipid peroxidation by flow cytometry. [file FEBS-292-6345-s001.pdf]

Supplementary Figures

Supplementary Figure 1

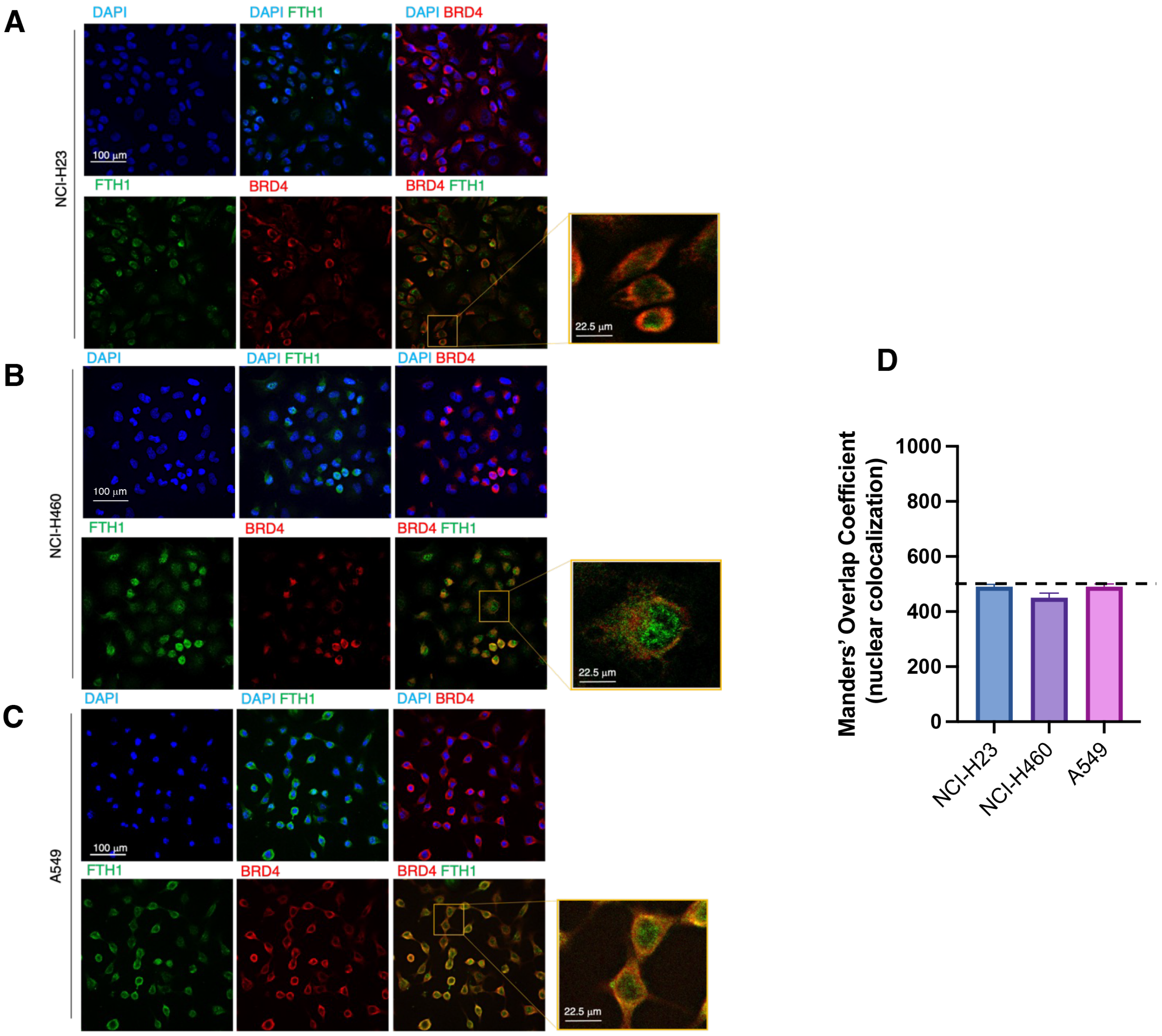

Supplementary Figure 1. Ferritin heavy chain does not interact with BRD4 in a panel of NSCLC cells. A. NHI-H23, **B.** A549, and **C.** NHI- H460 cells were grown on a coverslip, fixed with 4% paraformaldehyde and processed for double-label immunofluorescence with anti-FTH1 (green) and anti-BRD4 (red) antibodies. DAPI (blue) was used for nuclei staining. Images were collected using Thunder microscopy system as single z-stack images (63x); scale bar 100µm, and scale bar 22.5µm for magnified images. Representative images are shown. **D.** Graph showing levels of FTH1 and BRD2 colocalization in NHI-H23, NHI-H460 and A549 cell lines in nuclei, using Manders' overlap coefficient (MOC). MOC represent the percentage of BRD4 (red) that colocalized with FTH1 (green). Quantification rate was performed with >60 cells examined per cell line from 3 different experiments. For panels D-G, all results are mean ± SEM from three representative experiments. Data were statistically analyzed by Student's *t* test.

Supplementary Figure 2

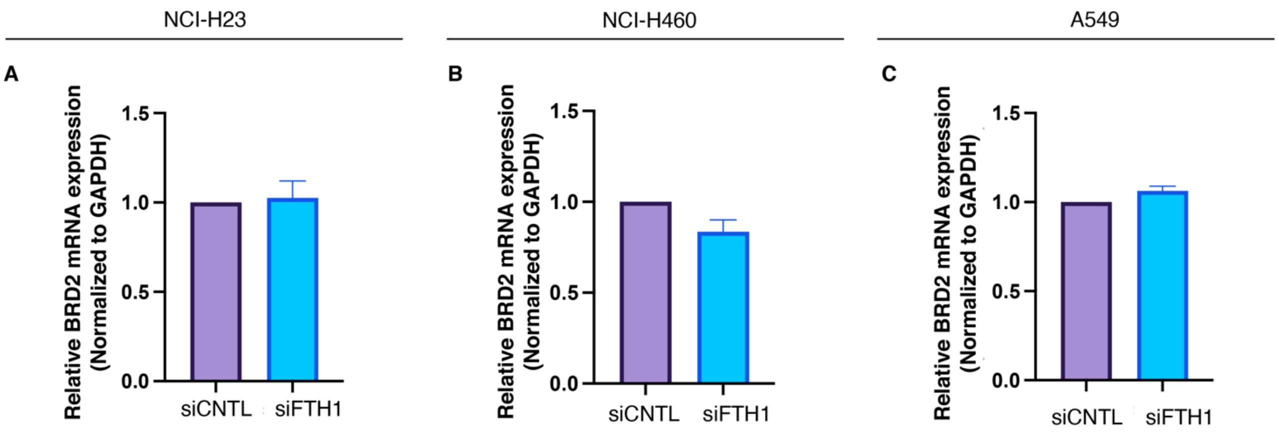

Supplementary Figure 2. Ferritin heavy chain is not involved in BRD2 mRNA alteration in NSCLC cells. A., B., and **C**., Total RNA was extracted from NCI-H23, NCI-H460, and A549 cells silenced for FTH1 or control, respectively, and analyzed by real-time PCR for the expression of BRD2. Results were normalized using *GAPDH* as the housekeeping gene. Data were statistically analyzed by Student's *t* test and are reported as mean values ± SEM of three independent experiments.

Supplementary Figure 3

A

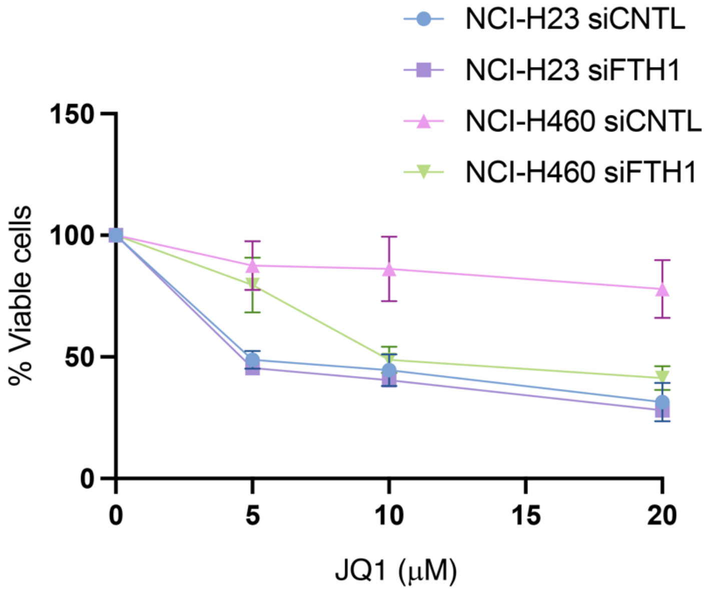

B

JQ1	
Cell lines	IC_{50} values (µM)
NCI-H23^{siCNTL}	5
NCI-H23^{siFTH1}	3.9
NCI-H460^{siCNTL}	ND
NCI-H460^{siFTH1}	12.6

Supplementary Figure 3. Cell viability analysis by Cell titer Glo in NCI-H23, and NCI-H460 cell lines 24 h after treatment with JQ1 at the indicated concentrations. **A.** Representative cell viability assay demonstrating dose-dependent growth inhibition and resistance in NSCLC cells treated with JQ1 determined by the Cell Titer Glo cell viability assay. IC_{50s} for NCI-H23^{siCNTL}, NCI-H23 ^{siFTH1} and NCI-H460^{siFTH1} cells respond to JQ1 whereas NCI-H460^{siCNTL} cells do not respond to JQ1. Results are reported as means of three independent experiments, each conducted in triplicate, and expressed as the percentage of viable siCNTL-transfected cells.

B) The Table on the right reports the IC_{50} values for the NCI-H23^{siCNTL}, NCI-H23^{siFTH1}, NCI-H460^{siCNTL}, and NCI-H460^{siFTH1} cells.

Supplementary Figure 4

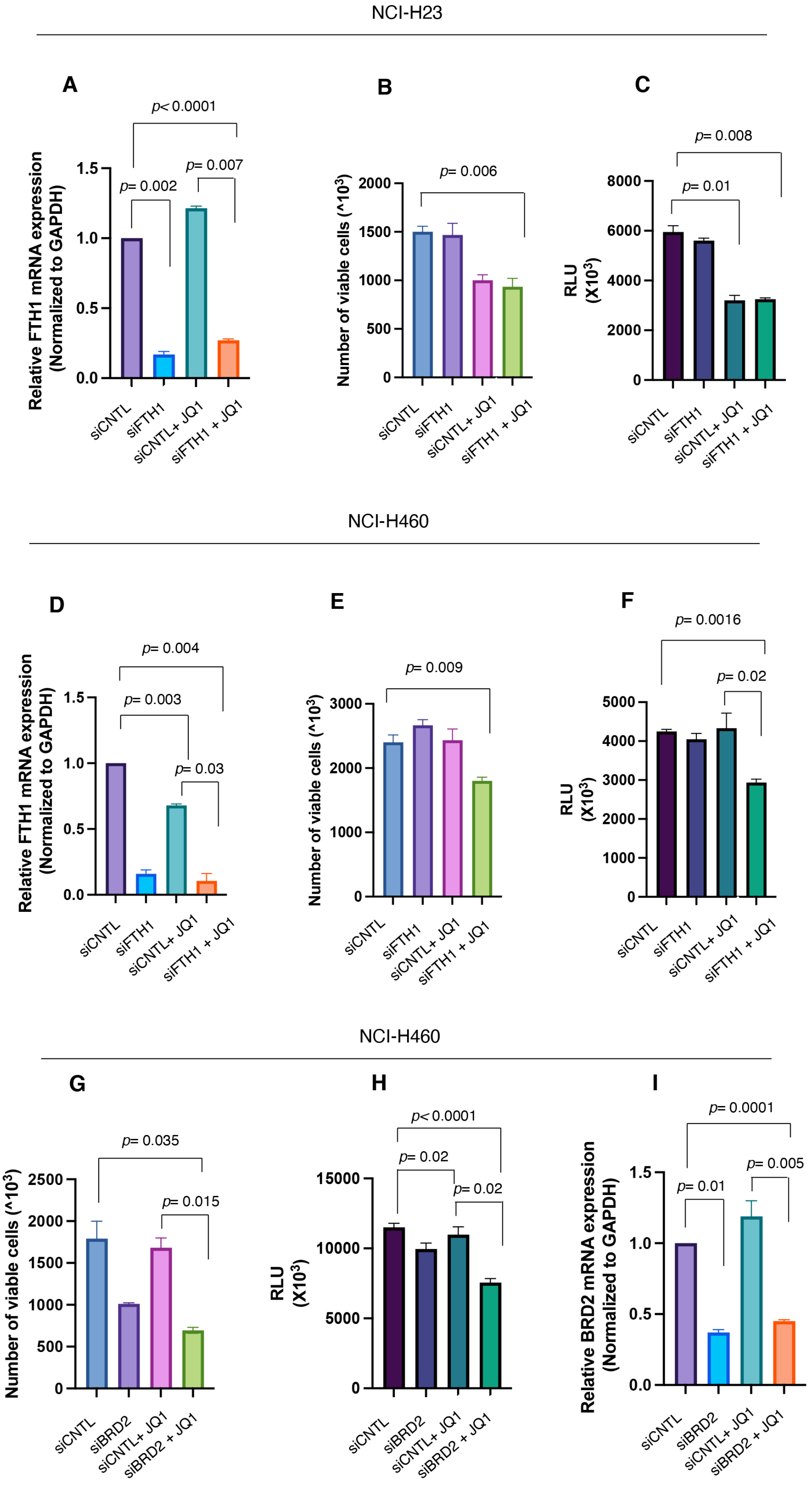

Supplementary Figure 4. FTH1 silencing combined with BET inhibitor JQ1 inhibits the growth of NCI-H460 JQ1-insensitive cells. The same effect is observed when BRD2 is knocked down. Cells (3 x 10^6) were transiently transfected with a pool of siFTH1or siRNA control (NCI-H23, NCI-H460). Twenty-four hours later, transfected cells were treated or not with JQ1 and lysed twenty-four hours later. **A.**, and **D.** Total RNA was extracted from NCI- H23^{siCNTL} and NCI-H23^{siFTH} cells with or without JQ1 treatment or NCI- H460^{siCNTL} and NCI- H460^{siFTH1} cells with or without JQ1 treatment, and analyzed by real-time PCR for the expression of *FTH1*. Results were normalized using *GAPDH* as the housekeeping gene. Data were statistically analyzed by Student's *t* test and are reported as mean values ± SEM of three independent experiments. Cell viability was determined in technical triplicate by **B.**, and **E**. Tripan Blue Dye exclusion assay and **C.** , and **F.**, CellTiter-Glo assay. The luminescence in relative light units (RLU) was plotted. Data shown are representative results of three independent experiments. Values (mean ± SEM; *n* = 3) are shown. Statistically significant difference according Student's *t* test.

NCI-H460 cells (3 x 10^6) were transiently transfected with a pool of siBRD2 or siRNA control. Twenty-four hours later, transfected cells were treated or not with JQ1 and lysed twenty-four hours later. Cell viability was determined in technical triplicate by **G.** Tripan Blue Dye exclusion assay and and **H.** CellTiter-Glo assay. The luminescence in relative light units (RLU) was plotted. Data shown are representative results of three independent experiments. Values (mean ± SEM; *n* = 3) are shown. Statistically significant difference according Student's *t* test. **I.** Total RNA was extracted from NCI- H460^{siCNTL} and NCI- H460^{siBRD2} cells with or without JQ1, and analyzed by real-time PCR for the expression of *BRD2*. Results were normalized using *GAPDH* as the housekeeping gene. Data were statistically analyzed by Student's *t* test and are reported as mean values ± SEM of three independent experiments.

Supplementary Figure 5

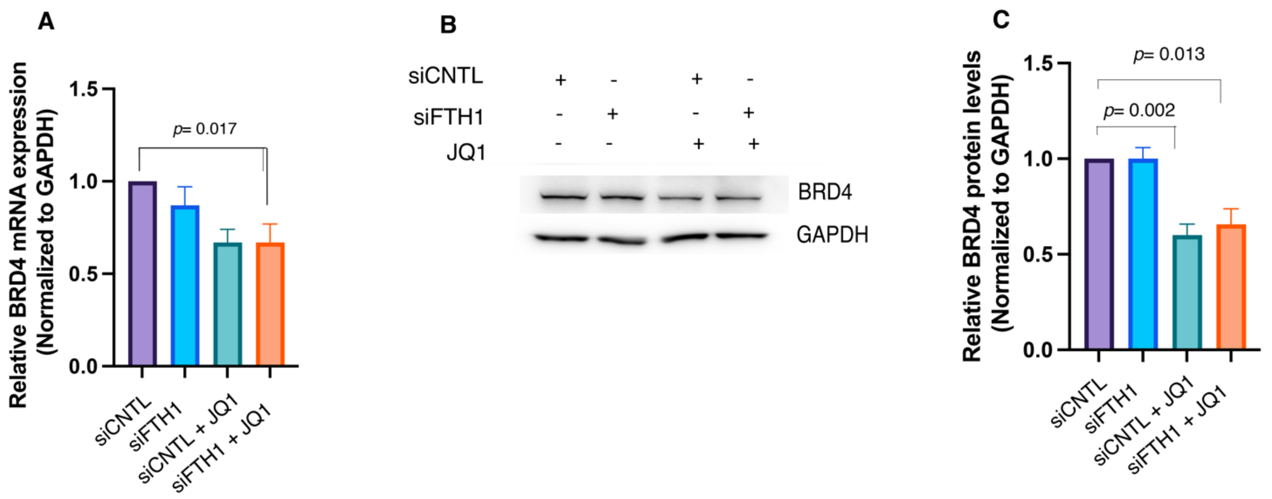

Supplementary Figure 5. Ferritin heavy chain does not influence BRD4 levels in NCI-H460 cells. A. Total RNA was extracted from NCI- H460^{siCNTL} and NCI- H460^{siFTH1} cells with or without JQ1 treatment, and analyzed by real-time PCR for the expression of *BRD4*. Results were normalized using *GAPDH* as the housekeeping gene. Data were statistically analyzed by Student's *t* test and are reported as mean values ± SEM of three independent experiments. NCI-H460 cells (3×10^6) were transiently transfected with a pool of siFTH1or siRNA control. Twenty-four hours later, transfected cells were treated or not with JQ1 and lysed twenty-four hours later. **B.** whole cell extracts (30 µg) of NCI-H460^{siCNTL} and NCI- H460^{siFTH1} cells w/o JQ1 treatment, were separated by 12% SDS-PAGE and analyzed by western blotting using anti-BRD4, and anti- GAPDH antibodies. The same cell lysate was also used for experiments in Figure 4A, and therefore we show the same anti-GAPDH western blot **C.** Densitometric values of BRD4 bands were normalized to GAPDH bands. Values (mean ± SE, *n* = 3) are shown. Statistically significant difference was calculated according to Student's *t* test.

Supplementary Figure 6

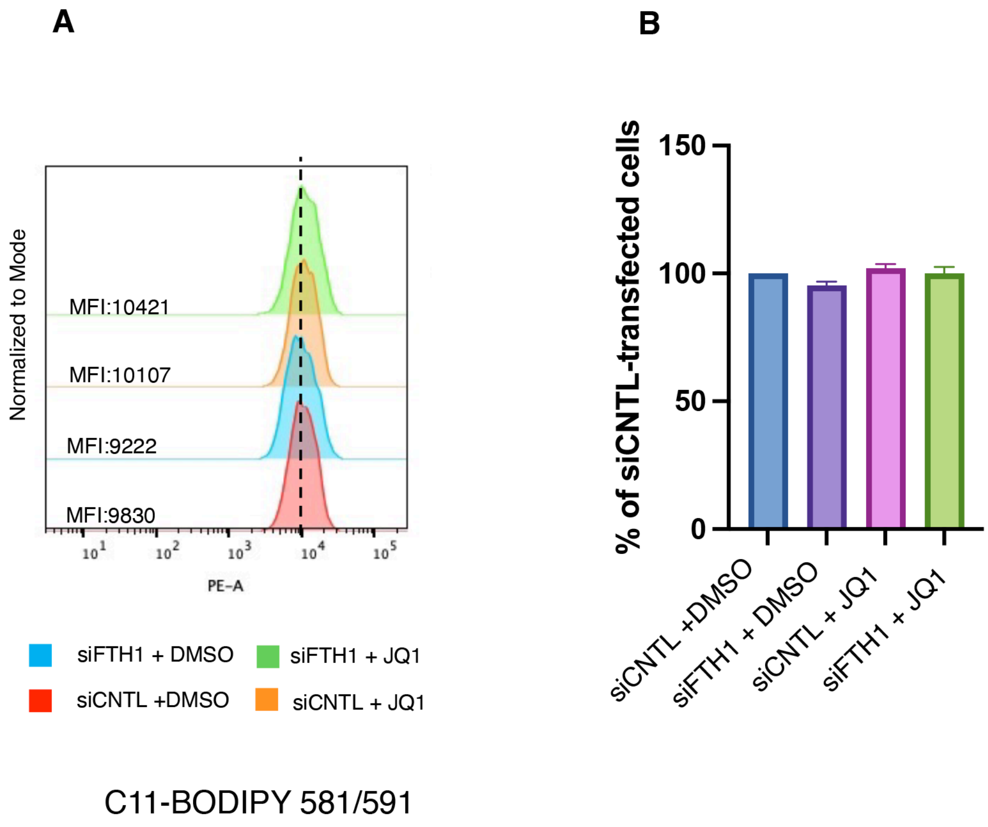

Supplementary Figure 6. Analysis of lipid peroxidation by flow cytometry. A. C11-BODIPY assay in NCI-H460 cells transiently transfected with a pool of siFTH1 or siRNA control, treated or not with JQ1(10µM) for 24h. The vertical dashed line indicates the cut-off relative to the control cells (siCNTL+DMSO). **B.** Bar diagram showing lipid peroxidation levels presented as the % of control (siCNTL-transfected cells). Data were statistically analyzed by Student's *t* test and are reported as mean values ± SEM of three independent experiments.
